# The ubiquitin-ligase TRAF6 and TGFβ type I receptor form a complex with Aurora kinase B contributing to mitotic progression and cytokinesis in cancer cells

**DOI:** 10.1016/j.ebiom.2022.104155

**Published:** 2022-07-16

**Authors:** Jie Song, Yang Zhou, Ihor Yakymovych, Alexej Schmidt, Chunyan Li, Carl-Henrik Heldin, Maréne Landström

**Affiliations:** a Department of Medical Biosciences, Pathology, Umeå University, SE-901 85 Umeå, Sweden; b Department of Medical Biochemistry and Microbiology, Science for Life Laboratory, Uppsala University, Box 582, SE-751 23 Uppsala, Sweden

**Keywords:** APPL1, AURKB, Cancer, Mitosis, TβRI, TRAF6

## Abstract

**Background:**

Transforming growth factor β (TGFβ) is overexpressed in several advanced cancer types and promotes tumor progression. We have reported that the intracellular domain (ICD) of TGFβ receptor (TβR) I is cleaved by proteolytic enzymes in cancer cells, and then translocated to the nucleus in a manner dependent on the endosomal adaptor proteins APPL1/2, driving an invasiveness program. How cancer cells evade TGFβ-induced growth inhibition is unclear.

**Methods:**

We performed microarray analysis to search for genes regulated by APPL1/2 proteins in castration-resistant prostate cancer (CRPC) cells. We investigated the role of TβRI and TRAF6 in mitosis in cancer cell lines cultured in 10% FBS in the absence of exogenous TGFβ. The molecular mechanism of the ubiquitination of AURKB by TRAF6 in mitosis and the formation of AURKB–TβRI complex in cancer cell lines and tissue microarrays was also studied.

**Findings:**

During mitosis and cytokinesis, AURKB–TβRI complexes formed in midbodies in CRPC and KELLY neuroblastoma cells. TRAF6 induced polyubiquitination of AURKB on K85 and K87, protruding on the surface of AURKB to facilitate its activation. AURKB–TβRI complexes in patient's tumor tissue sections correlated with the malignancy of prostate cancer.

**Interpretation:**

The AURKB–TβRI complex may become a prognostic biomarker for patients with risk of developing aggressive PC.

**Funding:**

Swedish Medical Research Council (2019-01598, ML; 2015-02757 and 2020-01291, CHH), the Swedish Cancer Society (20 0964, ML), a regional agreement between Umeå University and Region Västerbotten (ALF; RV-939377, -967041, -970057, ML). The European Research Council (787472, CHH). KAW 2019.0345, and the Kempe Foundation SMK-1866; ML. National Microscopy Infrastructure (NMI VR-RFI 2016-00968).

**Research in context**

**Evidence before this study**

TGFβ inhibits growth of normal epithelial cells and thereby protects against cancer development, but also promotes progression of advanced cancers by stimulation of epithelial-mesenchymal transition, which makes cancer cells more invasive. If and how TGFβ enhances tumor growth is not well understood. Proteolytic cleavage of the transmembrane TβRI generates a soluble intracellular domain (TβRI-ICD) in cancer cells. TβRI-ICD enters the cell nucleus in complex with endosomal proteins APPL1 and APPL2 in a manner dependent on the ubiquitin ligase TRAF6 and TGFβ stimulation. TβRI-ICD drives expression of pro-invasive genes, as well as of *transforming growth factor beta receptor 1* (*TGFBR1*) itself. AURKB is a key kinase of the chromosomal passenger complex (CPC), which is essential for proliferation of cancer cells. Aberrant and high expression of AURKB is frequent in prostate cancer as well as other cancer forms, while the precise mechanisms of regulation of *AURKB* expression and the role of AURKB kinase activity remain to be elucidated.

**Added value of this study**

By knocking down expression of *APPL1* and *APPL2, AURKB* was identified as a target gene for the APPL1/APPL2 regulated pathway in castration-resistant prostate cancer (CRPC) cells. TRAF6 was found to be autoubiquitinated during mitotic progression and to contribute AURKB activity through K63-linked polyubiquitination of AURKB on K85 and K87. AURKB formed a complex with APPL1 and TβRI during mitosis and cytokinesis in CRPC cells, as well as in neuroblastoma cells. APPL1 and TβRI were found to be required for proliferation of CRPC cells. High expression of AURKB and TβRI complexes visualized by *in situ* proximity ligation assay was present in clinical prostate cancer materials and correlated to poor prognosis. The expression of *AURKA* and *AURKB* was higher in CRPC of neuroendocrine type than in CRPC adenocarcinoma, consistent with the poor prognosis for patients with CRPC of neuroendocrine type.

**Implications of all the available evidence**

APPL1/APPL2 promoted expression of AURKB in CRPC cells. TRAF6 was autoubiquitinated and activated during mitotic progression, causing K63-linked polyubiquitination of AURKB on K85 and K87, which contributed to AURKB kinase activity and regulation of proliferation of CRPC cells. APPL1 and TβRI formed a complex with AURKB during mitosis and cytokinesis which was most prominent in midbodies. High number of AURKB and TβRI complexes were found in clinical materials of prostate cancer patients with high Gleason Score, indicative of a poor prognosis. The identification of complexes between AURKB and TβRI may be a useful predictive biomarker in tumor tissues from patients with aggressive prostate cancer.

Alt-text: Unlabelled box

## Introduction

Prostate cancer is the most common cancer diagnosed in men worldwide, particularly in the Western countries, causing about 375,000 deaths each year.^1^^,^^2^ Transforming growth factor β (TGFβ) determines cell fate and differentiation during embryogenesis and has important roles in several types of malignancies.^3^^,^^4^ The canonical TGFβ–Smad signaling pathway depends on the kinase activity of type I TGFβ receptor (TβRI), and involves the formation of Smad2, Smad3, and Smad4 complexes that regulate the transcription of certain genes, including *SERPINE1, Snail1*, and *metalloproteinase protein 2* (*MMP2*)*.*^4^, ^5^, ^6^ TβRI is cleaved in its extracellular domain by tumor necrosis factor-α-converting enzyme (TACE/ADAM17), resulting in a loss of growth inhibitory effects mediated by TGFβ mediated by Smad-proteins.^7^ Certain non-canonical TGFβ signaling pathways have been found to be regulated by the E3-ligase tumor necrosis factor receptor–associated factor 6 (TRAF6). This protein binds to TβRI and is activated upon ligand binding to receptors, in a receptor kinase-independent manner, promoting activation of the MAP kinase kinase kinase TGFβ-activated kinase 1.^8^, ^9^, ^10^ TRAF6 promotes activation of the phosphatidylinositol-3`-kinase (PI3K)-AKT pathway in response to insulin stimulation through K63-linked polyubiquitination of the endosomal protein APPL1 on K160,^11^ and in response to TGFβ stimulation by K63-linked polyubiquitination of the regulatory subunit p85α in the PI3K complex.^12^ TRAF6 also activates proteolytic enzymes, i.e. TACE/ADAM17 and presenilin 1 in the γ-secretase complex to release the intracellular domain (ICD) of TβRI. After ubiquitination of K178 by TRAF6, TβRI-ICD enters the nucleus, promoting transcription of pro-invasive genes and *TGFBR1*.^13^, ^14^, ^15^ We have recently shown that the endosomal adaptor proteins APPL1 and APPL2 associate with TβRI-ICD and enhance nuclear accumulation of TβRI-ICD in response to TGFβ stimulation, promoting invasiveness of prostate cancer cells *in vitro* through MMP2/MMP9*,* and showing a strong correlation with aggressiveness of human prostate cancers.^16^ In addition, the amount of TβRI-ICD in clear cell renal cell carcinoma (ccRCC) cells from patients has been shown to correlate with poor survival.^17^

The TGFβ signaling pathway has dual and pivotal roles in tumor progression. In normal cells and at early-stages of tumorigenesis, it acts as a tumor suppressor by inhibiting proliferation and inducing apoptosis.^3^^,^^18^ TGFβ inhibits proliferation of several cell types, including epithelial and endothelial cells, keratinocytes, and leukocytes, by downregulating expression of MYC and upregulating the expression of cyclin-dependent kinase inhibitors, including p15^INK4B^ and p21.^19^, ^20^, ^21^ However, in advanced cancers TGFβ promotes tumorigenesis by inducing epithelial-mesenchymal transition, facilitating tumor invasion and metastasis, and by suppression of the immune system.^21^, ^22^, ^23^

Despite these findings, little is known about the role of TGFβ in mitosis. Of interest, TGFβ can promote proliferation of certain mesenchymal and cancer cells, but the mechanism of growth stimulation is poorly understood. As a stimulator of proliferation, TGFβ induces expression of fibroblast growth factor 2 in human renal fibroblasts, and platelet-derived growth factor in glioma and osteosarcoma cells.^24^, ^25^, ^26^ In normal prostatic epithelial cells, TGFβ acts as a growth suppressor by inhibiting proliferation and inducing apoptosis, whereas in prostate cancer cells, which have lost sensitivity to TGFβ-induced growth arrest, TGFβ may promote tumor cell growth. For example, TGFβ stimulates cell proliferation in the prostate cancer cell line TSU-Pr1,^27^ and causes only transient inhibition of proliferation in the DU145 and PC-3 cell lines, while having no effect on proliferation of LNCaP prostate carcinoma cells.^28^ Interestingly, high expression of *TGFBR1* in several forms of cancers, including colorectal carcinoma, glioma, lung squamous carcinoma, pancreatic adenocarcinoma, stomach adenocarcinoma and invasive breast carcinoma, has been shown to correlate with poor survival.^29^

Aurora kinase A (AURKA) and B (AURKB) are overexpressed in many tumors, including breast, lung, pancreatic, ovarian, and prostate tumors.^30^, ^31^, ^32^ AURKB is a component of the chromosomal passenger complex (CPC), which also contains the inner centromere protein (INCENP), survivin, and borealin.^33^^,^^34^ AURKB binds to the conserved C-terminal IN-box region of INCENP,^35^ where a Thr-Ser-Ser (TSS) motif is located, which is phosphorylated by AURKB,^36^ contributing to AURKB activation and stabilization of the complex.^36^, ^37^, ^38^ The AURKB:INCENP complex has also been suggested to favor autophosphorylation of AURKB in trans, and a study of the crystal structure of AURKB revealed its form.^37^ In interphase, CPC localizes in the heterochromatin, and after a cell enters mitosis, AURKB phosphorylation of histone H3 at Ser10 (H3pS10) facilitates removal of CPC from the chromosome arms to the inner centromere.^33^^,^^34^^,^^39^ At anaphase onset, CPC is released from the chromosomes and re-localizes to the spindle midzone, where a phosphorylation gradient of AURKB is formed.^33^^,^^40^ During cytokinesis, CPC targets to the cleavage furrow and midbody. Because of the association between increased expression of AURKB in several different cancer types and poor prognosis, several AURKB inhibitors are being tested in clinical trials.^30^^,^^41^

## Methods

### Cell culture

The human prostate cancer cell line PC-3U (RRID:CVCL_0482) and the human neuroblastoma cell line KELLY^42^ which was purchased from Sigma-Aldrich (RRID: CVCL_2092) were grown in RPMI-1640, supplemented with 10% fetal bovine serum (FBS), 2 mM l-glutamine, and 100 units/ml penicillin and 0.1 mg/ml streptomycin.^43^ Immortalized wild-type mouse embryonic fibroblasts (MEFs) and MEFs deficient in TRAF6, were kind gifts from Jun-ichiro Inoue, and grown in Dulbecco's modified Eagle's medium containing 10% FBS, 4 mM l-glutamine, and 100 units/ml penicillin and 0.1 mg/ml streptomycin. Cell lines were tested negative for mycoplasma. For TGFβ stimulation experiments, TGFβ (5 ng/mL) was added to cells starved for 18 h in RPMI medium supplemented with 1% FBS. Transient transfection was performed with FuGENE® HD (Promega) according to the manufacturer's instructions. The PC3U cell line have been validated by Eurofins Genomics in July 2021.

### Antibodies used for immunoblotting

Antibodies against the following proteins were used for immunoblotting: APPL1 (Cell Signaling Technology Cat# 3858, RRID:AB_2056989), p-Aurora kinases (Thr288 in AURKA; Thr232 in AURKB; Thr198 in AURKC; the molecular masses of the proteins are 48 kDa, 40 kDa, and 35 kDa, respectively) (Cell Signaling Technology Cat# 2914, RRID:AB_2061631), cyclin B1 (Cell Signaling Technology Cat# 4135, RRID:AB_2233956), HA (Cell Signaling Technology Cat# 3724, RRID:AB_1549585 and Cat# 2367, RRID:AB_10691311), GFP (Cell Signaling Technology Cat# 2956, RRID:AB_1196615), GAPDH (Cell Signaling Technology Cat# 5174, RRID:AB_10622025), p38 (Cell Signaling Technology Cat# 8690, RRID:AB_10999090), survivin (Cell Signaling Technology Cat# 2808, RRID:AB_2063948), APPL2 (Santa Cruz Biotechnology Cat# sc-67403, RRID:AB_2056383), AURKB (Abcam Cat# ab2254, RRID:AB_302923), TRAF6 (Abcam Cat# ab40675, RRID:AB_778573), Flag (Sigma-Aldrich Cat# F9291, RRID:AB_439698), β-actin (Sigma-Aldrich Cat# A5441, RRID:AB_476744), β-tubulin (Sigma-Aldrich Cat# T0198, RRID:AB_477556 and Cell Signaling Technology Cat# 2146, RRID:AB_2210545), H3pS10 (Millipore Cat# 06-570, RRID:AB_310177), and TβRI (V22; Santa Cruz Biotechnology Cat# sc-398, RRID: AB_632493; the epitope of this specific antibody is located within the ICD of TβRI, as described before^13^). Horseradish peroxidase–coupled secondary antibodies were purchased from Dako and Protein-G Sepharose and ECL immunoblotting detection reagents from GE Healthcare. Pefabloc was obtained from Roche, PageRuler Prestained Protein Ladder was from Thermo Fisher Scientific.

### Protein analysis

Cells were washed twice with ice-cold phosphate-buffered saline (PBS) and lysed in ice-cold lysis buffer [150 mM NaCl, 50 mM Tris, pH 8.0, 0.5% (v/v) sodium deoxycholate, 1% (v/v) NP40, 10% (v/v) glycerol and protease inhibitors]. After centrifugation, the supernatants were collected and protein concentrations determined using the BCA Protein Assay Kit (Thermo Fisher Scientific). Equal amounts of protein from each total cell lysate were used for immunoprecipitation. Immunoprecipitated proteins were resolved by sodium dodecyl sulfate (SDS)-polyacrylamide gel electrophoresis (PAGE) on Mini-PROTEAN TGX gels (Bio-Rad), blotted onto nitrocellulose or polyvinylidene difluoride membranes and subjected to immunoblotting, as described previously.^16^

### *In vivo* ubiquitination assay

PC-3U cells were washed once in ice-cold PBS, collected in 1 ml ice-cold PBS, and then centrifuged at 300 × g for 5 min at 4°C. Non-covalent protein interactions were dissociated in freshly-made 1% SDS in PBS and by boiling for 10 min. Samples were diluted in 1.5 ml PBS containing 0.5% NP-40 with protease inhibitors. The samples were subjected to immunoprecipitation, followed by immunoblotting.^12^

### Immunofluorescence and microscope image acquisition

Other primary antibodies against the following proteins were used in immunofluorescence experiments: AURKB (Novus, Cat# NBP2-50039, RRID:AB_2895237), and p-Smad2 (Cell Signaling Technology Cat# 3108, RRID:AB_490941). Secondary antibodies were: donkey anti-rabbit Alexa Fluor 555 (Thermo Fisher Scientific Cat# A-31572, RRID:AB_162543), donkey anti-mouse Alexa Fluor 555 (Thermo Fisher Scientific Cat# A-31570, RRID:AB_2536180), goat anti-mouse Alexa Fluor 488 (Thermo Fisher Scientific Cat# A-11029, RRID:AB_2534088), and goat anti-rabbit Alexa Fluor 488 (Thermo Fisher Scientific Cat# A32731, RRID:AB_2633280). Immunofluorescence assays were performed as described previously.^16^ Briefly, cells were plated on coverslips, fixed in 4% paraformaldehyde for 30 min, and then treated with 0.2% Triton X-100 in PBS for 5 min and blocked with 10 mM glycine. Incubation with primary antibodies was performed for 1 h at room temperature, followed by washing in PBS and incubation with secondary antibodies. Photomicrographs were obtained using a confocal microscope LSM 710 (Carl Zeiss) with a 63 × /1.4 NA objective lens (Carl Zeiss). The images were acquired under oil immersion at room temperature, using Zen 2011 software.

### Plasmids and site-directed mutagenesis

pCR3-Flag-AURKB K106R kinase dead (KD) was a kind gift from Susanne Lens (Addgene Plasmid #108488; http://n2t.net/addgene:108488; RRID: Addgene_108488)^44^ and was used for context optimization and to generate a construct expressing the Flag-tagged wild-type AURKB protein by QuickChange Lightning MultiSite-Directed Mutagenesis kit (Agilent Technologies). The primers for mutagenesis were oJS5, oJS8, oJS17, and oJS18 (Figure S1a). Plasmids expressing altered Flag-AURKB (i.e., K85R, K87R, and K85/87R mutants) were generated by PCR mutagenesis, using oligo oJS9, oJS10, and oJS11, respectively. Similar approaches were employed to construct plasmids expressing the enhanced green fluorescent protein (EGFP)-fused to the wild-type AURKB, as well as the K85R, K87R, and K85R/K87R mutants, using pEGFP-AURKB K106R (KD) as the template for mutagenesis (Addgene Plasmid #108493; http://n2t.net/addgene:108493; RRID: Addgene_108493).^44^ Integration of tags and alterations of AURKB sequences were confirmed by DNA sequencing of the individual plasmids.

Plasmids carrying 6His-APPL1 and 6His-APPL2 (purchased from Thermo Fisher Scientific), were used as templates for mutagenesis to generate constructs producing transcripts that were tolerant of siRNA-induced gene silencing. The sequence of siRNA-resistance construct of APPL1 was 5’-AGAGAGATGGATTCAGACATA-3’, and the sequence of siRNA-resistance construct of APPL2 was 5’-CAGATTTATCTCACAGATAAC-3’. Alterations in APPL1 and APPL2 sequences were confirmed by DNA sequencing. YFP-APPL1-ΔN and GFP-APPL1-ΔC were kind gifts from Marta Miaczynska.^45^ pEGFPC1-human APPL1 was a gift from Pietro De Camilli (Addgene plasmid #22198; http://n2t.net/addgene:22198; RRID:Addgene_22198)^46^ and was used to generate constructs harboring BAR, PH, and PTB domains respectively, by QuickChange Lightning MultiSite-Directed Mutagenesis kit (Agilent Technologies). The primers for mutagenesis were oYZ86, oYZ87, oYZ88, oYZ91 and oYZ92 (Figure S1a). Alterations of APPL1 sequences were confirmed by DNA sequencing of the individual plasmids.

### siRNA transfection

On TARGET plus *APPL1* (No. 1 target sequence, 5′-GGAAAUGGACAGUGAUAUA-3′; No. 2 target sequence, 5′-GAUCUGAGUCUACAAAUUU-3′), *APPL2* (No. 1 target sequence, 5′-AGAUCUACCUGACCGACAA-3′; No. 2 target sequence, 5′-GCGGAAAAGAUGCGGGUGU-3′), *TGFBR1* siRNA (target sequence, 5’-CAUAUUGCUGCAAUCAGGA-3’), SMART pool *TRAF6* siRNA, and siGENOME non-targeting control siRNA #1 (target sequence, 5′-UAGCGACUAAACACAUCAA-3’) were obtained from Dharmacon Research. siRNA was transfected into cells using Oligofectamine Transfection Reagent (Thermo Fisher Scientific), according to the manufacturer's protocol.

### Total RNA extraction and microarray assay

After knockdown of *APPL1* and *APPL2*, total RNA was extracted from PC-3U cells using the RNeasy Mini Kit (Qiagen). RNA purity and integrity were evaluated with the Agilent RNA 6000 Nano Kit and Agilent 2100 Bioanalyzer (Agilent Technologies). Total RNA (500 ng) was used to generate a biotin-labeled antisense RNA target with the TargetAmp^TM^-Nano Labeling Kit for Illumina Expression Beadchip (Epicenter), following the manufacturer's protocol. RNA (750 ng) was hybridized to an Illumina Human HT-12 Beadchip array for 17 h. The chips were washed and stained with Cy3-streptavidin according to the manufacturer's instructions. Image data were acquired using the iScan system (Illumina). Microarray data were analyzed using GenomeStudio and DAVID Bioinformatics Resources 6.7 and verified by qRT-PCR.

### *In vitro* kinase assay

For *in vitro* kinase assay, HEK293T cells were transfected with vectors for Flag-tagged AURKB or its mutants K85R, K87R, and K85/87R, or the control empty pcDNA3 vector, using FuGENE® HD (Promega). Proteins were extracted in RIPA lysis buffer (150 mM NaCl, 0.1% Triton X-100, 0.5% sodium deoxycholate, 0.1% SDS, 50 mM Tris-HCl, pH 8.0, protease inhibitors (Roche)) and immunoprecipitated with anti-Flag antibody (Sigma-Aldrich Cat# F1804, RRID:AB_262044) and protein G Sepharose (Invitrogen). The beads were washed four times in RIPA buffer, and equilibrated in kinase buffer (15 mM MOPS, pH 7.2, 7.5 mM glycerol 2-phosphate, 15 mM MgCl_2_, 3 mM EGTA, 0.15 mM dithiothreitol).

The phosphorylation reaction was initiated by addition of substrate, histone H3 (1 μg) and ATP. In non-radioactive kinase assays, the concentration of ATP was 0.5 mM, while it was 5 µM in assays with 0.5 μCi [γ-32P] ATP (Perkin Elmer). For analyses by SDS-PAGE, reactions were stopped by addition of one-fifth volume of 6x SDS sample buffer, heated at 96 °C for 5 min and subjected to SDS-PAGE.

Phosphorylation of histone H3 was detected by immunoblotting with anti-phospho-histone H3 (Ser10) antibody (Millipore Cat# 06-570, RRID:AB_310177). Equal expression and loading were controlled by immunoblotting of the membranes with anti-histone H3 antibody (Cell Signaling Technology Cat# 4499, RRID:AB_10544537) and with anti-Flag antibody (Sigma-Aldrich Cat# F1804, RRID:AB_262044).

### Evaluation of cell number and cell death

Cell number was measured using the Cell Proliferation Kit I (MTT) from Roche or automated cell counter Countess™ from Thermo Fisher Scientific. Cell apoptosis was analyzed using Arthur™ after staining with the Tali™ apoptosis kit (Thermo Fisher Scientific).

### *In situ* proximity ligation assay (PLA)

For PLA brightfield, the tissue microarray (TMA; BioCat) was first deparaffinized, and then subjected to antigen retrieval and permeabilization. PLA was then performed using antibodies against AURKB (Novus, Cat# NBP2-50039, RRID:AB_2895237), K63-linked polyubiquitin (Abcam Cat# ab179434, RRID:AB_2895239) and TβRI (Santa Cruz Biotechnology Cat# sc-398, RRID: AB_632493) with Duolink Detection for Brightfield (Sigma-Aldrich). Images were acquired with Pannoramic 250 Flash, and PLA signals were analyzed using Duolink Image Tool software.

### Bioinformatics

Genes correlating with *TGFBR1* in prostate cancer were identified by calculating Pearson's correlation coefficients using log2 CPM (counts-per-million) normalized expression data of the TCGA PRAD cohort. All genes were ranked by their correlation to *TGFBR1*, and Gene Set Enrichment Analysis (GSEA) was performed using the R package clusterProfiler^47^ with the Hallmark gene sets of the Molecular Signatures Database (MSigDB).^48^ Thirty-four gene sets were enriched with an adjusted *p*-value of 0.05 or below.

RNA-seq expression data and clinical metadata from The Cancer Genome Atlas were downloaded using the Genomic Data Commons^49^ and the R package *TCGAbiolinks*,^50^ v. 2.16.4. The primary and secondary Gleason grades for each prostate tumor were obtained from the file PRAD_clindata.xls. Tumors were grouped based on their Gleason scores. The log2 CPM normalized expression values of each gene of interest were plotted per Gleason group using the R package *ggpubr*.^51^ The statistical significance of the expression difference was calculated using Student's t-test.

RNA-seq expression data and copy-number data for 49 castration-resistant prostate cancer (CRPC) samples from a published study^52^ were downloaded from the cBio Cancer Genomics Portal (http://cbioportal.org). Clinical data was obtained from the file data_clinical_sample.txt, expression data from data_RNA_Seq_expression_median.txt and the copy-number data from data_log2CNA.txt. The data were read and subjected to all further analysis using R, v. 4.0.2.^53^ The expression data were log2-transformed, and a row-normalized heatmap was plotted with the samples sorted by subtype and tumor location, and genes hierarchically clustered by their expression profile. The *RB1* copy-number status was defined as gain for an *RB1* log2 copy-number value of 0.4 or above, as a loss for a value of −0.4 or below, and copy neutral otherwise. Copy-number data were unavailable for three adenocarcinoma samples. The expression difference of the genes of interest in neuroendocrine vs adenocarcinoma CRPC groups along with Mann-Whitney U test *p*-values were visualized with box plots generated by the *ggboxplot* function of the R package *ggpubr*.^51^ Pearson's correlation between the expression of *TGFBR1* and other genes were calculated within neuroendocrine CRPC samples and adenocarcinoma CRPC samples.

### Statistics

The Student's t-test or Mann-Whitney U test were used to analyze differences between two independent groups as indicated in the figure legends. Values are expressed as the mean±standard error of the mean (SEM) or ±standard deviation (SD) of at least three independent experiments. *p*-values less than 0.05 were considered statistically significant. * *p* < 0.05, ** *p* < 0.01, ***p < 0.001.

### Ethics

The clinical materials (paraffin tissue microarrays) investigated in this study were purchased from BioCat.

### Role of funders

The funders have had no role in study design, data collection, data analyses, interpretation, or writing the manuscript or decision to publish it. ML has not been paid to write this article by a pharmaceutical company or other agency. The co-authors were not precluded from accessing data in the study, and they accept responsibility to submit for publication.

## Results

### APPL proteins regulate genes involved in proliferation and apoptosis

**Figure 1:**
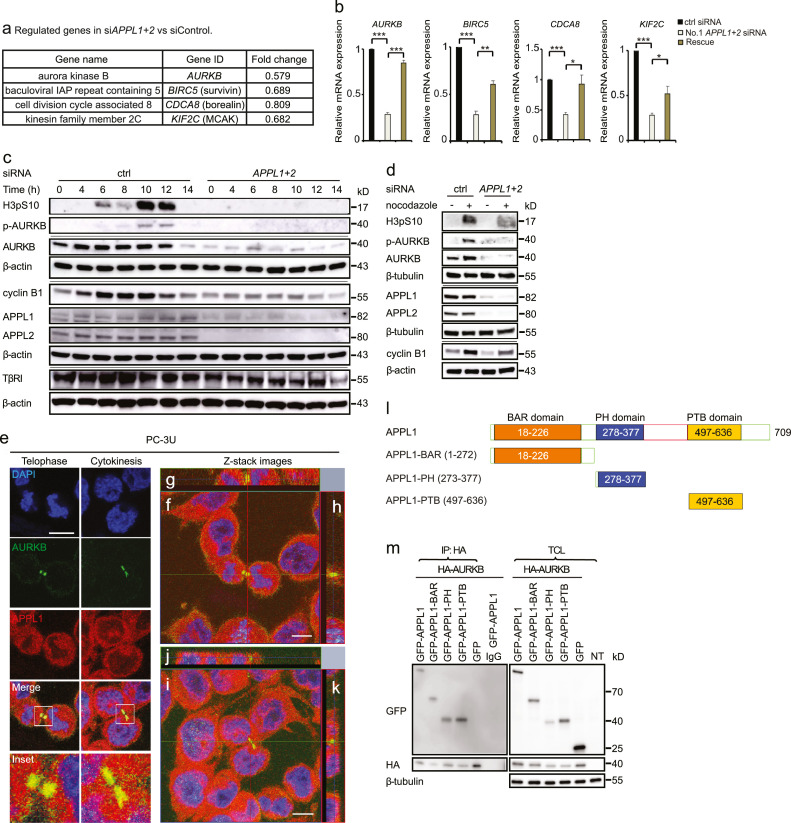
APPL1 and 2 promote *AURKB, BIRC5, CDCA8*, and *KIF2C* expression. (a) Human prostate cancer PC-3U cells were transfected with control or No. 1 *APPL1* and *APPL2* siRNA. RNA was extracted from cells, and microarray analysis was performed. (b) qRT-PCR analysis of the genes shown in panel a of cells treated with or without No. 1 *APPL1* and *APPL2* siRNA. Inhibition by siRNA was overcome by expressing siRNA-resistant constructs; N = 4, data presented as mean±SEM [Student's t-test, * *p* < 0.05, ** *p* < 0.01, ***p < 0.001]. (c) PC-3U cells were synchronized with a double thymidine block and treated with No. 1 *APPL1* and *APPL2* siRNA. Cells were released and cell lysates were prepared at different times, and subjected to immunoblotting. (d) PC-3U cells were transfected with or without No. 1 *APPL1* and *APPL2* siRNA, incubated with nocodazole for 12 h, and analyzed by immunoblotting. (e) Immunofluorescence and confocal imaging showing co-localization of AURKB (green) and APPL1 (red) during telophase and cytokinesis. (f-k) Orthogonal views (XY, XZ and YZ) of two Z-stack images of panel e. (f, i) XY view (z-projection). (g, j) XZ view. (h, k) YZ view. Scale bar, 20 µm. (l) Schematic representation of the APPL1 protein and mutants. (m) PC-3U cells transiently transfected with HA-AURKB and different APPL1 domains as indicated, were synchronized and then subjected to immunoprecipitation with an antibody against HA and immunoblotting using a GFP antibody. Non-transfected (NT).

We have found that TβRI undergoes proteolytic cleavage in cancer cells in a manner dependent on TRAF6 and the active γ-secretase complex leading to the generation of TβRI-ICD.^13^^,^^14^ Moreover, the endosomal adaptor proteins Adaptor Protein, Phosphotyrosine Interacting with PH Domain and Leucine Zipper 1 (APPL1) and APPL2 are required for the nuclear accumulation of TβRI-ICD in response to TGFβ stimulation of cells. These chains of events promote invasiveness of prostate cancer cells *in vitro* and were also found to correlate with aggressiveness of human prostate cancers.^16^ To investigate in further detail the target genes of the nuclear APPL1–TβRI-ICD-complex, we performed microarray analyses to assess the effects on gene expression of knocking down *APPL1/2* (Figure 1a). Among the affected genes in *APPL1/2* knockdown cells, we observed decreased expression of genes encoding proteins involved in cell proliferation and apoptosis, i.e., components of the CPC [AURKB, survivin (encoded by *BIRC5*), and borealin (encoded by *CDCA8*)] and their downstream substrate, mitotic centromere-associated kinesin (encoded by *KIF2C*) (Figure 1a).^33^^,^^34^^,^^54^ No effect was observed on expression of *INCENP.*

The microarray data was verified using quantitative real-time PCR (qRT-PCR). Specifically, we confirmed that the expression of *AURKB, BIRC5, CDCA8*, and *KIF2C* was decreased in cells transfected with two different *APPL1*/*2* small interfering (si)RNAs (Figure 1b and S1b). Re-expression of the wild-type APPL1/2 protein from siRNA-resistant constructs overcame the inhibition by *APPL1*/*2* siRNA to a significant extent (Figure 1b).

Since AURKB functions in the CPC complex, we determined the level of proteins and protein-protein interactions during mitotic progression of cells grown in 10% FBS or as specified below. Using immunoblotting, reduced AURKB and survivin protein levels were observed in the *APPL1/2* knockdown cells (Figure S1c). To examine whether the AURKB expression level is related to APPL1/2 proteins, we used a double thymidine block to synchronize PC-3U cells and then released them into the normal medium with 10% FBS to follow cell cycle progression. When the cells were treated with *APPL1/2* siRNA, expression of AURKB and phosphorylation of its substrate H3 on S10, was dramatically decreased in the cell cycle (Figure 1c). The expression of cyclin B1 and TβRI was notably decreased after silencing the expression of APPL1/2 (Figure 1c). The reduced expression of TβRI in cells treated with *APPL1/2* siRNA is consistent with previous reports that nuclear TβRI-ICD promotes its own expression.^14^^,^^55^ In confirmation of these findings, cells that were arrested at the G2/M phase by nocodazole treatment also showed decreased levels of AURKB and H3pS10 (Figure 1d). Interestingly, using immunofluorescence microscopy and z-stack imaging analyses, APPL1 was found to co-localize with AURKB in the midbody (Figure 1e-k).

We previously reported that APPL1 is required for nuclear accumulation of TβRI-ICD and that the C-terminal part of APPL1 binds to TβRI.^16^ On the basis of these findings, we investigated the effect of N- and C-terminal deletions of APPL1 on the levels of AURKB. Indeed, the expression of a C-terminally deleted APPL1 mutant suppressed the level of AURKB, whereas an N-terminal deletion mutant did not have such an effect (Figure S1d). Moreover, by co-immunoprecipitation experiments, we found that AURKB associated with all three domains of APPL1, i.e. the BAR, PH, and PTB domains (Figure 1l- m).^45^^,^^56^ Taken together, these findings support the notion that APPL1 associates with and regulates the expression of AURKB, and that the expression of TβRI, which is reliant on nuclear TβRI-ICD,^14^^,^^55^ is cell cycle dependent.

### TβRI associates with AURKB in the midbody during mitosis and cytokinesis

**Figure 2:**
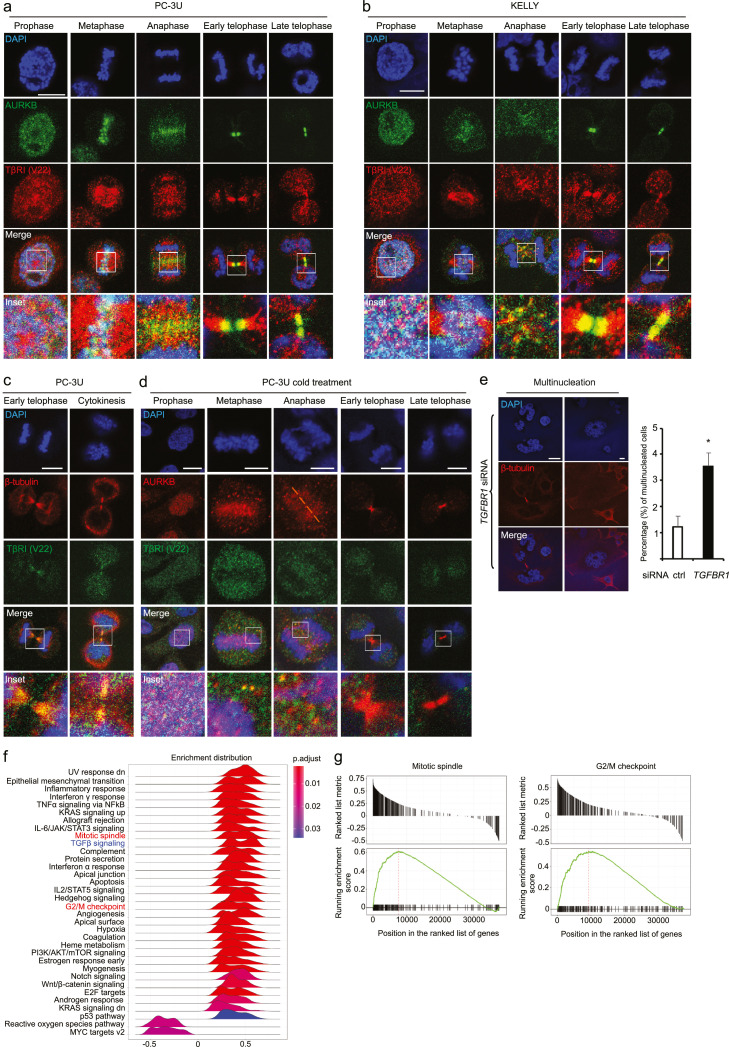
TβRI co-localizes with AURKB during mitosis. (a-c) Immunofluorescence experiments showing co-localization of AURKB (green) and TβRI (V22, red) during mitosis in human prostate cancer (PC-3U) (a) and human neuroblastoma (KELLY) (b) cells, and of TβRI (V22, green) and β-tubulin (red) throughout the PC-3U mitosis (c). Scale bar, 20 µm. (d) Decreased co-localization of TβRI and AURKB after treatment of PC-3U cells on ice for 30 min. Scale bar, 20 µm. (e) Multinucleated cells were counted after knockdown of *TGFBR1*. Data presented as mean±SEM, N=3 [Student's t-test, *p < 0.05]. Scale bar, 20 µm. (f) Gene Set Enrichment Analysis (GSEA) of genes ranked by their correlation with *TGFBR1* expression yielded 34 significantly enriched gene sets (adjusted *p*-value ≤ 0.05 and the *p*-values are adjusted using the Benjamini-Hochberg procedure). The ridge-plot shows the distribution of correlation coefficients of the core enriched genes, i.e., genes which contribute most to the enrichment of the gene set. The gene sets are ordered by normalized enrichment score. Color indicates the adjusted *p*-value. (g) GSEA plots of the hallmark mitotic spindle (left) and G2/M checkpoint (right) gene sets show their strong association with *TGFBR1*-correlated genes. The upper panels show the correlation coefficients and position of the gene set genes within the ranked list of all genes, and the lower panels show the running enrichment score.

We observed that APPL1 interacts with AURKB (Figure 1e-k and 1m) and forms a complex with TβRI.^16^ Since the expression of TβRI is cell cycle dependent, we next investigated whether TβRI also associates with CPC during mitosis and cytokinesis. Immunostaining experiments performed in PC-3U prostate cancer cells and KELLY neuroblastoma cells revealed that TβRI co-localized with AURKB in the midzone, as well as in the midbody (Figure 2a-b). We detected a partial co-localization between TβRI and survivin during telophase (Figure S2a), and TβRI and β-tubulin clearly co-localized in the midbody (Figure 2c).

APPL1 has been reported to transport TβRI-ICD from endosomes to the nucleus via microtubules.^16^ We therefore investigated whether intact microtubules are important for the TβRI localization; no interaction between AURKB and TβRI was seen in the midbody when microtubules were depolymerized by cold treatment (Figure 2d).^57^ Dynamic microtubules were also important for localization of AURKB during anaphase, which is consistent with a previous report.^58^ Furthermore, knockdown of *TGFBR1* led to multinucleation (Figure 2e), giving further support to the possibility of an important function for TβRI during cell division. Moreover, expression of *TGFBR1* was strongly correlated with mitotic spindle and G2/M checkpoint gene sets in prostate cancer (Figure 2f-g).

No p-Smad2 was found to localize in the midbody (Figure S2b), indicating that the canonical TGFβ signaling pathway is not active there. Taken together, these results suggest that TβRI and AURKB co-localize in the midbody and that this co-localization depends on an intact microtubule cytoskeleton.

### TRAF6 promotes polyubiquitination of AURKB on K85 and K87

**Figure 3:**
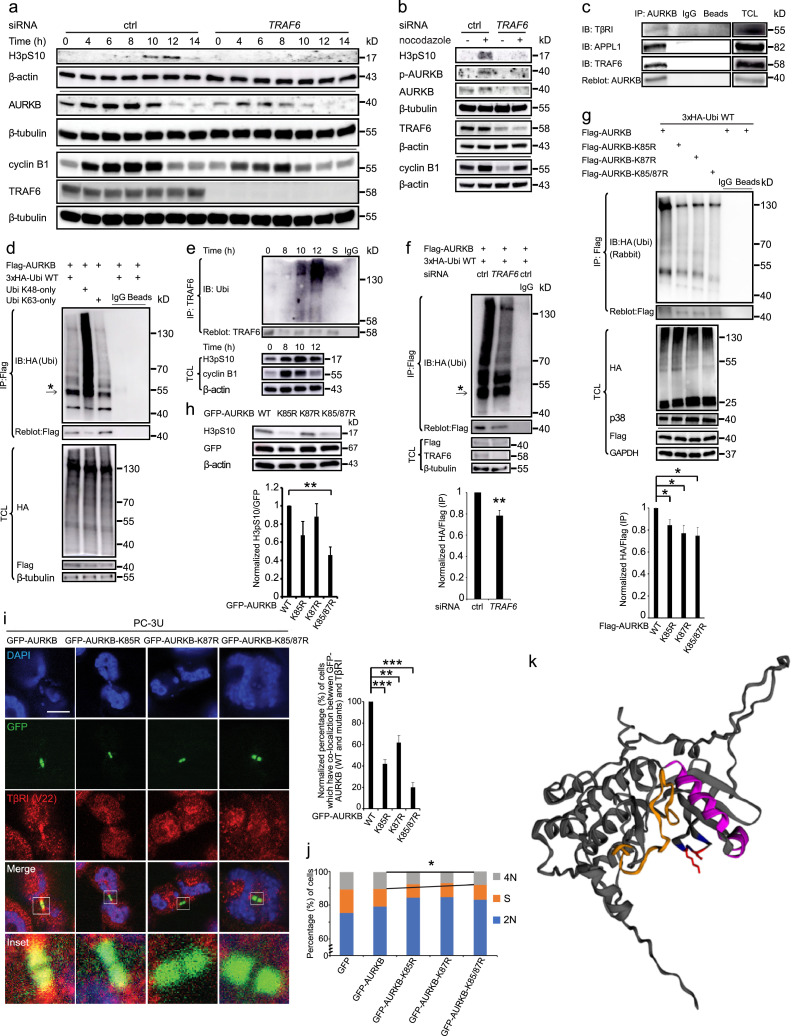
TRAF6 mediates K63-linked polyubiquitination of AURKB. (a-b) PC-3U cells were treated with or without *TRAF6* siRNA, synchronized with a double thymidine block and subjected to analysis by immunoblotting (IB) after different time periods (a), with or without incubation for 12 h with nocodazole (b). (c) Lysates of synchronized PC-3U cells were immunoprecipitated (IP) with an AURKB antibody, followed by immunoblotting with antibodies against TβRI, APPL1 and TRAF6. (d) Lysates of synchronized PC-3U cells transfected with Flag-AURKB and HA-tagged wild-type (WT) or mutated ubiquitin, were subjected to immunoprecipitation using a Flag antibody, followed by immunoblotting using an HA antibody. Arrow points to heavy immunoglobulin chain. (e) PC-3U cells were synchronized with a double thymidine block, released to fresh media with 10% FBS, harvested at the indicated times, and then subjected to *in vivo* ubiquitination assay. S is short for starvation (f). Lysates of synchronized PC-3U cells treated with or without *TRAF6* siRNA were subjected to immunoprecipitation using a Flag antibody, followed by immunoblotting using an HA antibody. Arrow points to heavy immunoglobulin chain. Data presented as mean±SEM, N=3 [Student's t-test, ** *p* < 0.01] (g) Lysates of synchronized PC-3U cells transfected with HA-tagged WT ubiquitin and Flag-tagged WT or mutant AURKB, were subjected to immunoprecipitation using a Flag antibody, followed by immunoblotting using an HA rabbit antibody. Data presented as mean±SEM, N=3 [Student's t-test, * *p* < 0.05]. (h) PC-3U cells were transfected with WT or mutant GFP-AURKB, and then subjected to immunoblotting with an antibody against H3pS10. Data presented as mean±SEM, N=3 [Student's t-test, ** *p* < 0.01]. (i) PC-3U cells were transfected with WT or mutant GFP-AURKB, then stained with TβRI (red). n=20, N=3, data presented as mean±SEM [Student's t-test, ** *p* < 0.01, ***p < 0.001]. (j) PC-3U cells were transfected with WT or mutant GFP-AURKB, then stained with Hoechst 33342. N=3 [Student's t-test, * *p* < 0.05]. (k) Schematic illustration of the kinase domain of AURKB. The structure file AF-Q96GD4-F1-model_V2pdb of human AURKB was downloaded from alphafold.ebi.ac.uk and uploaded to EzMol interface 2.1 (Imperial College London, UK) for visualization and depiction. The activation loop, the alpha C helixes (amino acid residues 110 to 131; alphaC´ (aa 110 to aa 115) and alphaC (aa 118 to 131)), K85 and K87, and G84, G86, G98, together with their corresponding side residues, are depicted in orange, magenta, red and blue, respectively. Note that the side chain of K85 and K87 (red) protrudes out from the kinase domain.

Next, we investigated the role of the ubiquitin E3-ligase TRAF6 for the expression of AURKB. We observed that *TRAF6* knockdown by siRNA led to decreased expression of both H3pS10 and AURKB during the cell cycle, as demonstrated by immunoblotting (Figure 3a-b). AURKB was found to associate with TβRI, APPL1 and TRAF6, as determined by a co-immunoprecipitation assay (Figure 3c).

AURKB has been reported to undergo ubiquitination, which is important for its re-localization from centromeres to microtubules^59^ and for its involvement in chromatin de-condensation and nuclear envelope formation.^60^ We found that AURKB underwent both K48-linked and K63-linked polyubiquitination when PC-3U cells were arrested in mitosis (Figure 3d). We also investigated if TRAF6 could be autoubiquitinated and activated during mitotic progression after release from double thymidine block. The endogenous TRAF6 was autoubiquitinated during 10-12 h after PC-3U cells were released from double thymidine block, i.e., at the time when AURKB is active (Figure 3e),^61^ consistent with current knowledge that autoubiquitination of TRAF6 enables its own catalytic activity.^62^

Knockdown of *TRAF6* by siRNA in PC-3U cells suppressed polyubiquitination of AURKB (Figure 3f). Immunostaining also revealed that endogenous TβRI co-localized with AURKB in a TRAF6-dependent manner in both PC-3U and MEF cell lines (Figure S3a-b). The consensus sequence for ubiquitination by TRAF6, i.e. -(hydrophobic)-K-(hydrophobic)-X-X-(hydrophobic)-(polar)-(hydrophobic)-(polar)-(hydrophobic), in which K is the ubiquitinated amino acid residue and X is any other residue,^63^ is found in AURKB (^84^GKGKFGNVYL), and is conserved among different species (Figure S3c); moreover, AURKB was reported to be ubiquitinated on K85 and K87 in a global search for ubiquitinated proteins.^64^ To investigate if K85 or/and K87 in AURKB is/are ubiquitinated and, if so, its functional consequence(s), we generated mutants in which these residues were mutated to arginine residues; we found that the ubiquitination of AURKB was indeed decreased in these mutants (Figure 3g). Interactions between TRAF6 and the AURKB mutants K85R, K85/K87R, and to lesser extent K87R, were also decreased compared to the interaction with wild-type AURKB, as determined by a co-immunoprecipitation assay (Figure S3d). Moreover, H3pS10 was decreased in cells overexpressing the AURKB K85/87R double mutant (Figure 3h), suggesting that ubiquitination of AURKB affects its kinase activity or capability to phosphorylate its substrate H3pS10. As K85 and K87 are localized in the glycine-rich loop of AURKB, which binds ATP, we investigated the AURKB mutants by *in vitro* kinase assay. Both the single mutants and the double mutant K85/87R were found to incorporate radioactive phosphate (Figure S3e), demonstrating that these mutations did not interfere with binding of ATP. To investigate whether AURKB mutants are intrinsically defective in kinase activity, an *in vitro* kinase assay using recombinant histone H3 as substrate was performed. All AURKB wild-type and mutants, except the kinase dead K106R mutant, which served as control for the experiment, could phosphorylate histone H3 at S10, thereby demonstrating kinase intrinsic activities of the AURKB mutants (Figure S3f).

Of interest, TβRI did not localize to midbodies when cells overexpressed the AURKB mutants (Figure 3i), suggesting that ubiquitination of AURKB is required for the recruitment of TβRI in midbodies. Double AURKB mutant (K85/87R)–expressing cells showed less 4N DNA content, compared to wild-type, supporting the biological relevance of polyubiquitination of AURKB on K85 and K87 during replication of the cells (Figure 3j). Both AURKA and AURKB contain a glycine rich loop which harbors K141&K143 and K85&K87 respectively and the loop locates in the beginning of the kinase domain (Figure S3g). A 3D structure of AURKB shows that K85 and K87 (red) locates in the protruding lysine side chains in the vicinity of the activation loop (orange) and alpha C-helixes (magenta) (Figure 3k). Overall, these results support the notion that TRAF6 is autoubiquitinated during mitotic progression and that TRAF6-mediated ubiquitination of AURKB on K85/K87 contributes to its kinase activity and controls the localization of TβRI in the midbody during cell division.

### Expression of *AURKB* and AURKB-TβRI complex formation correlate with poor prognosis in prostate cancer

**Figure 4:**
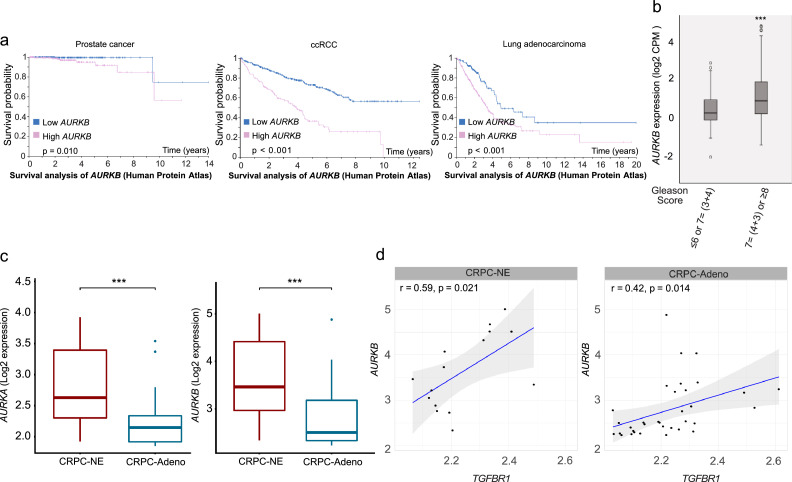
The expression of *AURKB* correlates with poor prognosis in different cancers. (a) Kaplan–Meier plots illustrating the effects on the survival of patients of low vs. high expression of *AURKB* in prostate cancer, ccRCC, or lung adenocarcinoma. Representative images were obtained from the Human Protein Atlas, based on data from the TCGA Pan Cancer Atlas database. (b) Expression of *AURKB* in the primary prostate tumors in TCGA differed between Gleason groups [Student's t-test, ***p < 0.001]. Tumors were grouped based on their Gleason scores. (c) The expression of *AURKA* and *AURKB* in CRPC-NE and CRPC-Adeno [Mann-Whitney U test, ***p < 0.001]. (d) The expression of *AURKB* and *TGFBR1* was correlated in both CRPC-NE and CRPC-Adeno. Pearson correlation analysis was used for data analysis.

Of note, high expression of *AURKB* mRNA also correlated with poor prognosis in prostate cancer, ccRCC, and lung adenocarcinoma (Figure 4a). *AURKB* expression correlated with the degree of malignancy of prostate cancer, as determined by the Gleason score, based on histopathological scoring in prostate cancer samples (a higher Gleason score indicates more aggressive disease) (Figure 4b). It has been reported that in several lung cancer and breast cancer cell lines, loss of *RB1* makes cells hyperdependent on AURKB for their survival.^65^ We therefore investigated the expression of *RB1* and *AURKB* in prostate cancer tissues. We found that *RB1* is deleted in 10% of prostate cancers (Figure S4a) and intriguingly, that expression of *AURKB* is negatively correlated with *RB1* in prostate cancer (Figure S4b). To further investigate expression of genes of interest in different prostate cancer types, we performed bioinformatics analysis using public database (Figure 4c-d and S4c-d).^52^ The expression of both *AURKA* and *AURKB* was higher in CRPC-neuroendocrine (CRPC-NE) than in CRPC-adenocarcinoma (CRPC-Adeno), consistent with the observation that CRPC-NE patients have a poor prognosis (Figure 4c).^66^^,^^67^ Furthermore, the expression of *AURKB*, correlated to the expression of *TGFBR1* in both CRPC-NE and CRPC-Adeno (Figure 4d). The relative expression of *TGFBR1, AURKA, AURKB, TRAF6, VPS4A/B* and *APPL1/2* in CRPC-NE and CRPC-Adeno including both primary tumors and metastases is also shown (Figure S4c). Interestingly, the expression of *APPL1* and *AURKA* correlated with *TGFBR1* in CRPC-NE but not in CRPC-Adeno (Figure S4d).

**Figure 5:**
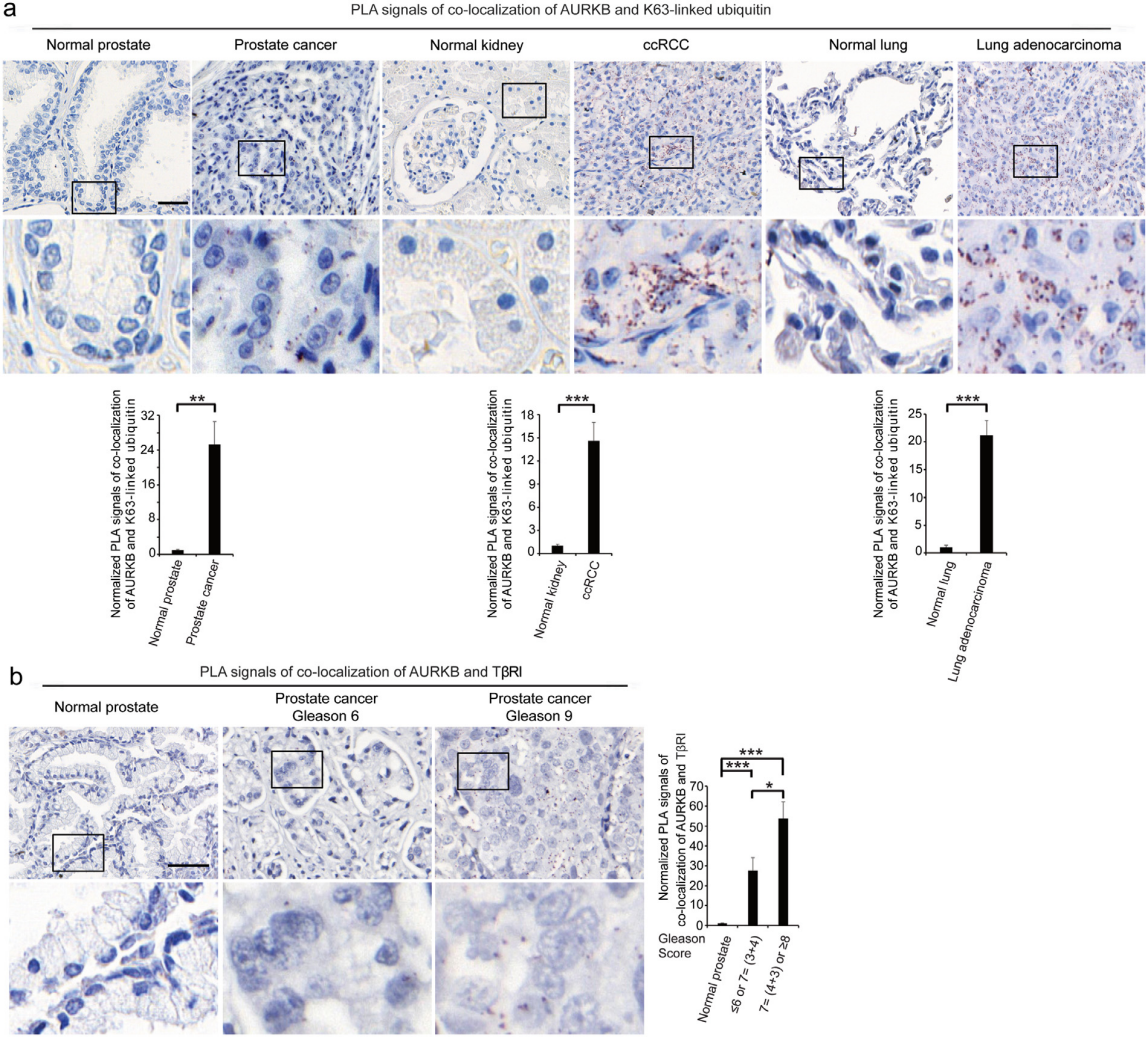
AURKB is ubiquitinated in different cancers and forms a complex with TβRI in prostate cancer. (a) *In situ* PLA was performed on TMAs to investigate the co-localization of AURKB and K63-linked ubiquitin (brown dots). The numbers of normal prostates, kidneys, and lungs were 22, 24, and 23, respectively. The numbers of prostate cancers, ccRCC, and lung adenocarcinoma were 41, 38, and 32, respectively. Quantification shows mean±SEM [Student's t-test, **p < 0.01, ***p < 0.001]. (b) The association between AURKB and TβRI in prostate cancer TMA of patient materials (brown dots) was determined by *in situ* PLA. A total of 29 patients with low Gleason scores and 28 patients with high Gleason scores were included. The number of normal prostates was 23. Quantification shows mean±SEM [Student's t-test, *p < 0.05, ***p < 0.001]. Scale bar, 50 μm.

To investigate the importance of AURKB and TβRI for cancer progression, we next determined their activity, expression, and complex formation in clinically derived samples. By using an *in situ* PLA, we investigated whether K63-linked polyubiquitination of AURKB could be visualized in tissues from patients with prostate cancer, ccRCC or lung adenocarcinoma. We observed a high number of K63-linked polyubiquitinated AURKB molecules in all three cancer types compared with normal tissues (Figure 5a). Moreover, we also identified a significantly higher number of AURKB and TβRI complexes in sections from patients with aggressive prostate cancer compared to those from patients with less aggressive disease; in normal prostate tissues almost no signals were observed (Figure 5b and S4e).

### APPL proteins, TβRI and TRAF6 affect cell growth and survival

TβRI associates with the endocytic adaptor protein APPL1, which has a role in cell proliferation and survival.^16^^,^^45^^,^^56^^,^^68^ Because the interaction between APPL1 and TβRI is important during cancer progression,^16^ we investigated whether APPL proteins affect proliferation or survival of PC-3U cells. For this purpose, we used the MTT (3-(4,5-dimethylthiazol-2-yl)-2,5-diphenyltetrazolium bromide) assay, which measures relative cell number. The results showed that knockdown of *APPL1/2* led to a decrease in cell numbers, suggesting that APPL1/2 is needed for cell proliferation or viability (Figure S5a).

To further investigate the possible role of APPL1/2 in cell survival, we quantified apoptotic cells and found more of them in *TGFBR1* and *APPL1/2* knockdown cell cultures than in controls (Figure S5b). For comparison, we also investigated the role of APPL1/2 in the cellular response to epidermal growth factor (EGF), which promotes cell proliferation and facilitates nuclear translocation of APPL proteins.^45^ Knockdown of *APPL1*/*2* with siRNA resulted in reduced cell numbers when compared with PC-3U control cells treated with EGF only (Figure S5c), suggesting that APPL proteins are important for proliferation or survival of EGF-stimulated cells, consistent with the observation of increased *APPL1* gene expression and protein expression during the initiation and progression of prostate cancer.^16^^,^^69^ We also observed reduced cell numbers in *TRAF6* and *TGFBRI* knockdown cultures of PC-3U cells, suggesting that TRAF6 and TβRI are required for cell proliferation or survival (Figure S5d). These findings are in line with previous reports that TRAF6 has oncogenic properties, as it is amplified in lung carcinoma^70^ and in aggressive prostate cancer.^29^^,^^52^^,^^71^ Increased expression of *TGFBRI* has been observed in several cancer forms, including aggressive breast carcinoma, and to correlate with poor prognosis.^29^^,^^72^ Moreover, knockdown of *TRAF6* in PC3 cells leads to reduced tumor growth *in vivo*,^73^ and knockdown of *TRAF6* has also been shown to reduce growth of pancreatic cancer cells *in vivo*.^74^

## Discussion

We have previously identified a signaling pathway in cancer cells, in which TβRI undergoes proteolytic cleavage in a TRAF6-dependent manner, generating TβRI-ICD which enters the nucleus when TβRI is polyubiquitinated by TRAF6 on residue K178.^13^, ^14^, ^15^ We also reported earlier that APPL1 interacts with TβRI-ICD via C-terminus of APPL1 and that the complex translocates to the nucleus via microtubules in a TRAF6-dependent manner.^16^ Once in the nucleus, TβRI-ICD induces the expression of *TGFBR1* and other genes by binding to their promoter regions.^14^ In this report, *AURKB* was identified as a target gene for the APPL1/APPL2-dependent pathway in CRPC cells *in vitro*. TRAF6 was found to be autoubiquitinated in CRPC cells during mitotic progression and to contribute to AURKB kinase activity through K63-linked polyubiquitination on K85/87 in a conserved glycine rich loop of AURKB. Moreover, we report that APPL1 and TβRI-ICD formed a complex with AURKB during mitosis and cytokinesis in CRPC cells. In addition, knockdown of *APPL1, TRAF6* or *TGFBR1* inhibited proliferation or survival of CRPC cells, suggesting that they are required for growth of CRPC *in vitro*.

Based on our current findings and our previous reports, we propose that TβRI-ICD acts together with AURKB to take part in regulation of mitosis and cytokinesis in a TRAF6-dependent manner, and TRAF6 causes polyubiquitination of AURKB on K85 and K87, as discussed further below. Mitosis is an extraordinary complex and highly controlled biological process, in which members of the Aurora kinase family have been shown to be required for chromosomal segregation.^41^^,^^75^^,^^76^ Aurora kinase family members AURKA, AURKB and AURKC, have key functions to control mitotic progression in normal cells and are frequently overexpressed and dysregulated in several forms of cancer, including prostate cancer, contributing to progression of the disease.^76^^,^^77^ The N- and C-terminal domains of Aurora kinases are conserved to a high degree^76^ and are folded in a closed formation when they reside in an inactive form.^78^ The N-terminal domains of Aurora kinases are thought to be important for their subcellular localizations. In the beginning of kinase domains of Aurora kinases harbor the glycine rich loop with the conserved GKGK-motif (Figure S3c, S3g) which binds ATP; this part of the molecule is flexible and in close contact with the activation loop (Figure 3k).^79^ K106 is crucial for activation of AURKB and mutation on this residue (K106R) results in inactivation of AURKB.^61^ Our data shows that TRAF6-induced K63-linked polyubiquitination on K85 and K87 in the beginning of kinase domain of AURKB occurs during mitotic progression of cancer cells when AURKB is active (Figure 3). K85 and K87 of AURKB and are exposed on the surface and in close contact with the two alpha-C-helixes and the activation loop of the molecule (Figure 3k and Figure S3g).

The AURKB structure adopts an active kinase formation upon autophosphorylation on T232, in the activation loop of AURKB.^80^ For AURKA it has been shown that phosphorylation in the catalytic domain occurs in an asymmetric dimer when one molecule acts as the active kinase and the other molecule is the substrate.^81^ Similar mechanisms for transactivation of a dimeric AURKB has been reported by Elkins,^37^ Azeez^38^ together with their colleges. Binding of INCENP to the N-terminal domain of AURKB contributes in an allosteric manner to induction of its active conformation as a kinase, and similar activation of AURKC has been reported,^38^ while allosteric activation of AURKA involves it's binding to TPX2.^76^ The partially active AURKB next phosphorylates INCENP in a conserved TSS-motif which results in full activation of AURKB.^37^^,^^80^ Our data propose that TRAF6-induced K63-linked polyubiquitination of AURKB on K85 and K87 might contribute to allosteric activation and stabilization of the AURKB complex formation in its active form (Figure 3), a notion that is supported by our finding that double mutation of K85 and K87 suppressed the kinase activity of AURKB and the fact that ubiquitination of enzymes could result in their activation due to an allosterically induced conformation of their 3D structure as well as to stabilize it's active conformation.^82^ Since the glycine rich loop is involved in ATP-binding, an alternative possibility would be that mutation of K85 and K87 perturbed ATP-binding and thereby inhibited the kinase activity of AURKB. However, mutation of the two lysine residues did not prevent autophosphorylation of AURKB (Figure S3e), which is consistent with the previous publication from Bose and colleagues,^83^ suggesting that this possibility is unlikely. Moreover, mutations of acceptor lysines K85 and K87 in AURKB did not affect its intrinsic kinase activity (Figure S3f). The glycine-rich sequence of AURKB is flexible and interacts with the catalytic site of the kinase.^84^ It is therefore possible that ubiquitination in this region changes its conformation and thereby facilitates kinase activity of AURKB, as discussed in detail above. An alternative possibility would be that polyubiquitination of AURKB on K85 and K87 facilitates its dimerization and promotes autophosphorylation on T232, which has been shown to occur in trans in a dimeric AURKB:INCENP complex.^37^^,^^38^^,^^85^ Importantly, TRAF6 was found to be autoubiquitinated, which is consistent with its activation,^62^ during mitotic progression (Figure 3e) at the same time as AURKB is active, in agreement with our hypothesis that active TRAF6 has impact on AURKB to regulate proliferation of cancer cells.

**Figure 6:**
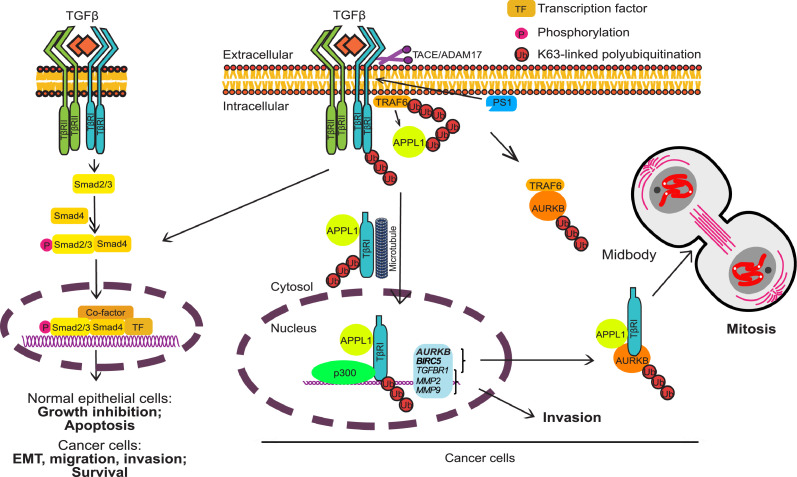
Schematic illustration of the TβRI-ICD signaling pathway and its involvement in mitotic progression. The non-canonical pathway in which TβRI undergoes proteolytic cleavage by TACE/ADAM17 and presenilin 1 in the activated γ-secretase complex, generates an intracellular domain (TβRI-ICD). The endosomal protein APPL1/2 and intact microtubules are required for the nuclear translocation of TβRI-ICD. In the nucleus, TβRI-ICD forms a complex with the transcriptional co-activator p300 and promotes expression of pro-invasive genes, *TGFBR1, MMP2/MMP9*, as well as *AURKB* and *BIRC5* (encoding survivin). During cell division, TβRI-ICD and APPL1 form a complex with AURKB. TRAF6 promotes K63-linked polyubiquitination of AURKB on K85 and K87 during mitosis, which together with TβRI-ICD are required for proper cell division.

By confocal imaging, we found that APPL1 and AURKB, as well as AURKB and TβRI, colocalized in midbodies during mitosis and cytokinesis. The co-localization of AURKB and TβRI is dependent on TRAF6 (Figure S3a-b). Moreover, by co-immunoprecipitation AURKB was shown to interact with APPL1, TβRI and TRAF6 (Figure 3c). AURKB was found to bind to all three domains of APPL1 (Figure 1m), while TβRI binds to the C-terminal of APPL1.^16^^,^^56^^,^^68^ It is possible that these interactions are dynamic during mitotic progression and cytokinesis, and the precise constitution of these complexes over time remains to be determined. However, our data suggest that AURKB and TRAF6 associate during mitotic progression to contribute to AURKB activity, and that during late telophase and cytokinesis APPL1, AURKB and TβRI localize in midbodies. Moreover, TβRI localization to midbodies was dependent on K63-linked polyubiquitination on K85 and K87 of AURKB, suggesting that TβRI associates with ubiquitinated AURKB (Figure 6).

Earlier work has shown that knockdown of *AURKB* in LNCaP, a human androgen-dependent prostate cancer cell line, does not affect tumor cell survival. In contrast, knockdown of *AURKB* in the more aggressive, androgen-independent PC3 cell line, results in apoptosis *in vitro* and reduced tumor growth in a xenograft nude mouse model *in vivo*,^86^ suggesting an important role for AURKB in androgen-independent prostate cancer cells. A previous study described AURKB-related tumor-promoting and pro-survival effects in CRPC.^77^ This result, and the current findings that the APPL1–TβRΙ-ICD pathway controls AURKB expression and that TβRΙ interacts with AURKB, support the notion that TβRI promotes cell proliferation in part through its role during cytokinesis and cell division. Thus, the growth-inhibitory effect transduced by the canonical TβRI–Smad signaling pathway in normal epithelial cells,^3^^,^^5^^,^^21^ is distinct from the role of TβRI-ICD in complex with AURKB during mitotic progression and cytokinesis, as reported herein. The observation that knockdown of *TGFBR1* led to multinucleation of cancer cells (Figure 2e), underscores the functional role of TβRI in cytokinesis of cancer cells.

AURKB is frequently overexpressed in various cancers, including prostate cancer.^30^ Errors in mitosis can lead to genome instability, which is an important hallmark of tumorigenesis.^87^ As noted, Aurora kinases are involved in multiple steps of mitosis, including centrosome maturation, bipolar spindle assembly, chromosome condensation, alignment, and cytokinesis.^76^ Because of their specific roles in regulating mitosis, they are target candidates in cancer treatment, with inhibitors being tested in clinical trials.^30^^,^^31^^,^^41^ Higher expression of *AURKB* also indicated poorer patient survival and more aggressiveness of prostate cancer (Figure 4a-b). Furthermore, in prostate cancer patients *TGFBR1* expression was highly correlated with mitotic spindle and G2/M checkpoint (Figure 2f-g). Moreover, the expression of *AURKA* and *AURKB* was higher in CRPC of the neuroendocrine type than in CRPC adenocarcinoma (Figure 4c), consistent with the poor prognosis for patients with CRPC of the neuroendocrine type.^66^^,^^67^ The amount of TβRI and AURKB complexes were more frequently observed in sections from prostate cancer patients with high Gleason score, which indicates more aggressive disease (Figure 5b). In summary, our data supports the idea that AURKB and TβRI form a functional complex during cell mitosis and cytokinesis to take part in cell proliferation and that TRAF6-induced ubiquitination of AURKB plays an important role, since the AURKB K85/87R mutant did not recruit TβRI to midbodies (Figure 3i).

Taken together, the findings presented in this study demonstrate a previously unknown function of TβRI in regulating cancer cell proliferation, i.e., through interaction with AURKB when the cells enter mitosis. This function is clearly distinct from the well-known function of TβRI as an upstream regulator of transcriptional responses via the canonical Smad signaling pathway, in response to TGFβ. TRAF6, which associates with TβRI,^8^ causes polyubiquitination of AURKB on specific residues (K85/87), thereby contributing to AURKB activity as measured by H3pS10 (Figure 6). The identification of a key function for the TβRI–AURKB complex in the mitosis and cytokinesis of cancer cells provides a basis for developing biomarkers for aggressive cancers that depend on this pathway.

## Contributors

J.S., Y.Z., I.Y. and C.Y.L. performed the experiments. AS has performed structure modelling of AURKB. J.S. and M.L. have verified the underlying data. J.S., Y.Z., A.S., C.H.H., and M.L. wrote, reviewed and/or revised the manuscript. All authors read and approved the final version of the manuscript.

## Data sharing statement

Datasets and additional documents generated, analyzed or used during the current study are available from corresponding author upon reasonable request. Microarray data is available on: https://www.ncbi.nlm.nih.gov/geo/query/acc.cgi?acc=GSE206243.

## Declaration of interests

J.S. and M.L. have submitted an international patent application No. PCT/EP2022/063820. M.L. is a founder, shareholder and board member of the company MetaCurUm Biotech AB that develops TβRI-based cancer therapies and biomarkers. The other authors declare no competing financial interests.

## Appendix Supplementary materials

Image, application 1

Image, application 2

Image, application 3
