# Supplementary material for: The ubiquitin-ligase TRAF6 and TGFβ type I receptor form a complex with Aurora kinase B contributing to mitotic progression and cytokinesis in cancer cells
Source: eBioMedicine. 2022 Jul 16;82:104155. doi: 10.1016/j.ebiom.2022.104155 (PMC9386726; doi:10.1016/j.ebiom.2022.104155)
Supplement: Supplementary file 1 [file mmc1.docx]

Supplementary material

Table of contents

Figure S1.

Figure S2.

Figure S3.

Figure S4.

Figure S5.

Original blots.

**
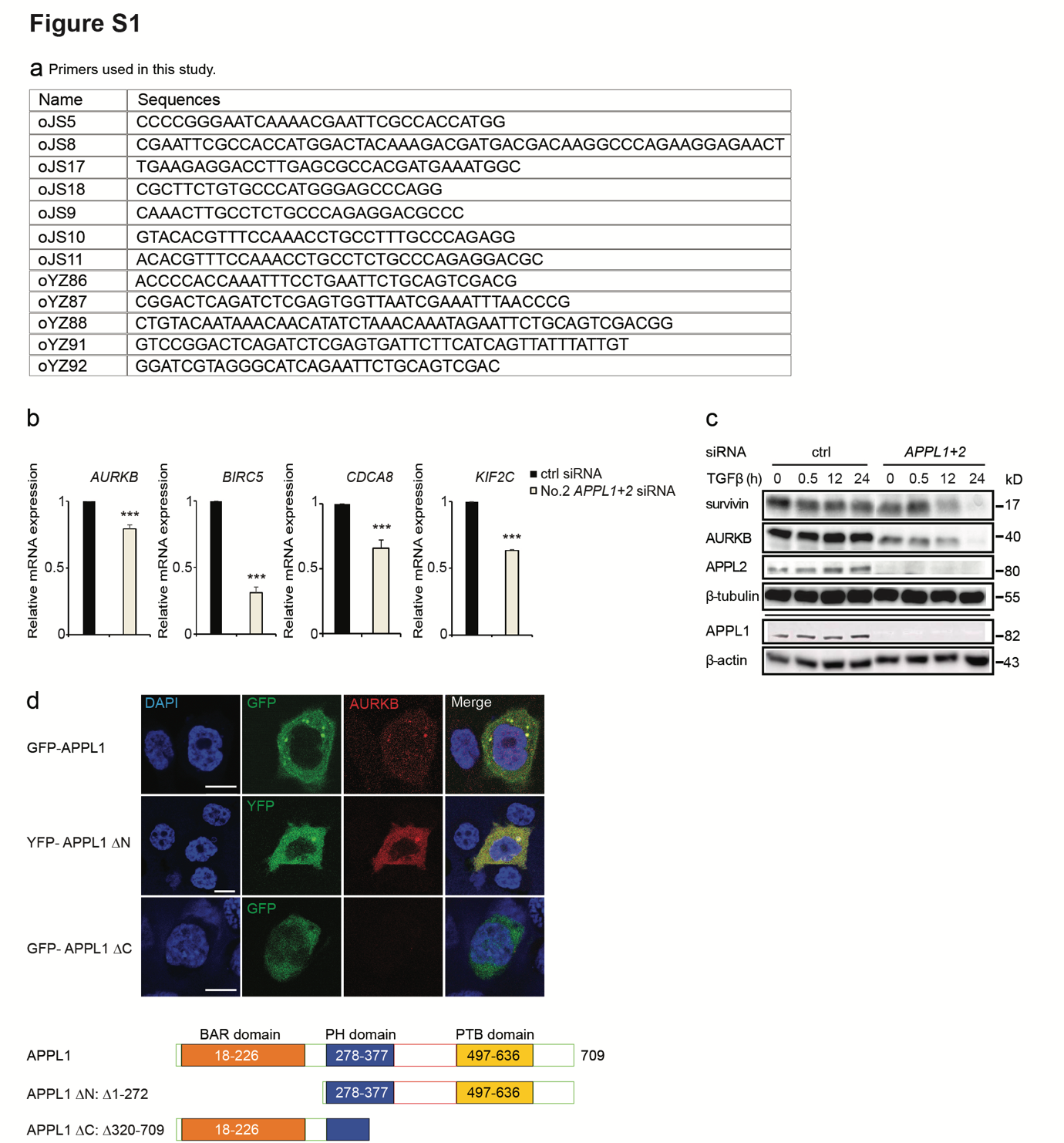
**

**Figure S1.** Effects of the expression of APPL1/2 on the expression of mRNA of *AURKB*, *BIRC5*, *CDCA8* and *KIF2C*, and proteins of survivin and AURKB. (a) The primers used in this study to generate AURKB and APPL1 plasmids are shown. (b) qRT-PCR was performed to validate the microarray results of Figure 1b using a second pair of siRNAs (No. 2; N=3). Data are presented as mean±SD [Student’s t-test, ***P<0.001]. (c) Expression of survivin and AURKB was evaluated by immunoblotting in PC-3U cells treated or not with No.1 *APPL1/2* siRNA and TGFβ. (d) PC-3U cells were transfected with full-length GFP-APPL1, yellow fluorescent protein (YFP)-APPL1-∆N, or GFP-APPL1-∆C and then stained with AURKB (red). The green channel was selected to show both GFP and YFP. Scale bar, 20 µm. A schematic representation of the APPL1 protein and mutants is included.


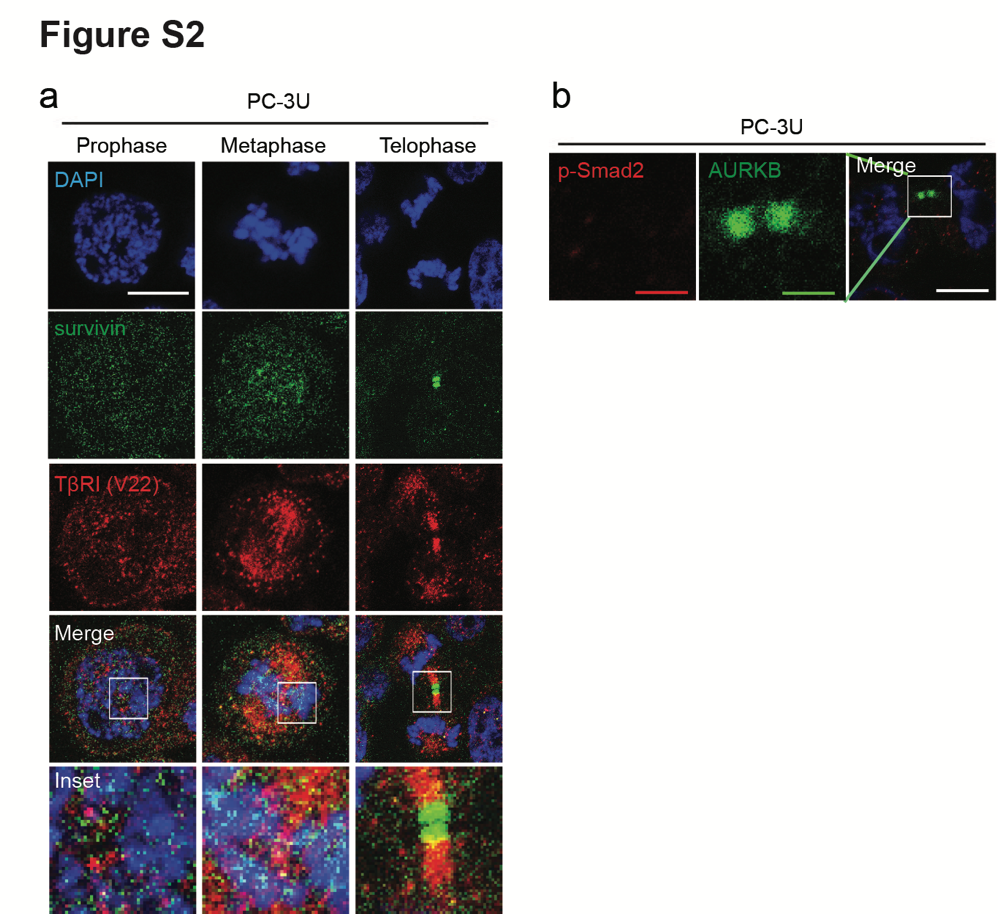


**Figure S2**. Characterization of TβRI and p-Smad2 in CPC during mitosis. (a) Localization of survivin (green) and TβRI (V22, red) in PC-3U cells throughout the mitosis. (b) PC-3U cells were stained with antibodies against p-Smad2 (red) and AURKB (green). Red and green scale bar, 5 µm; white scale bar, 20 µm.


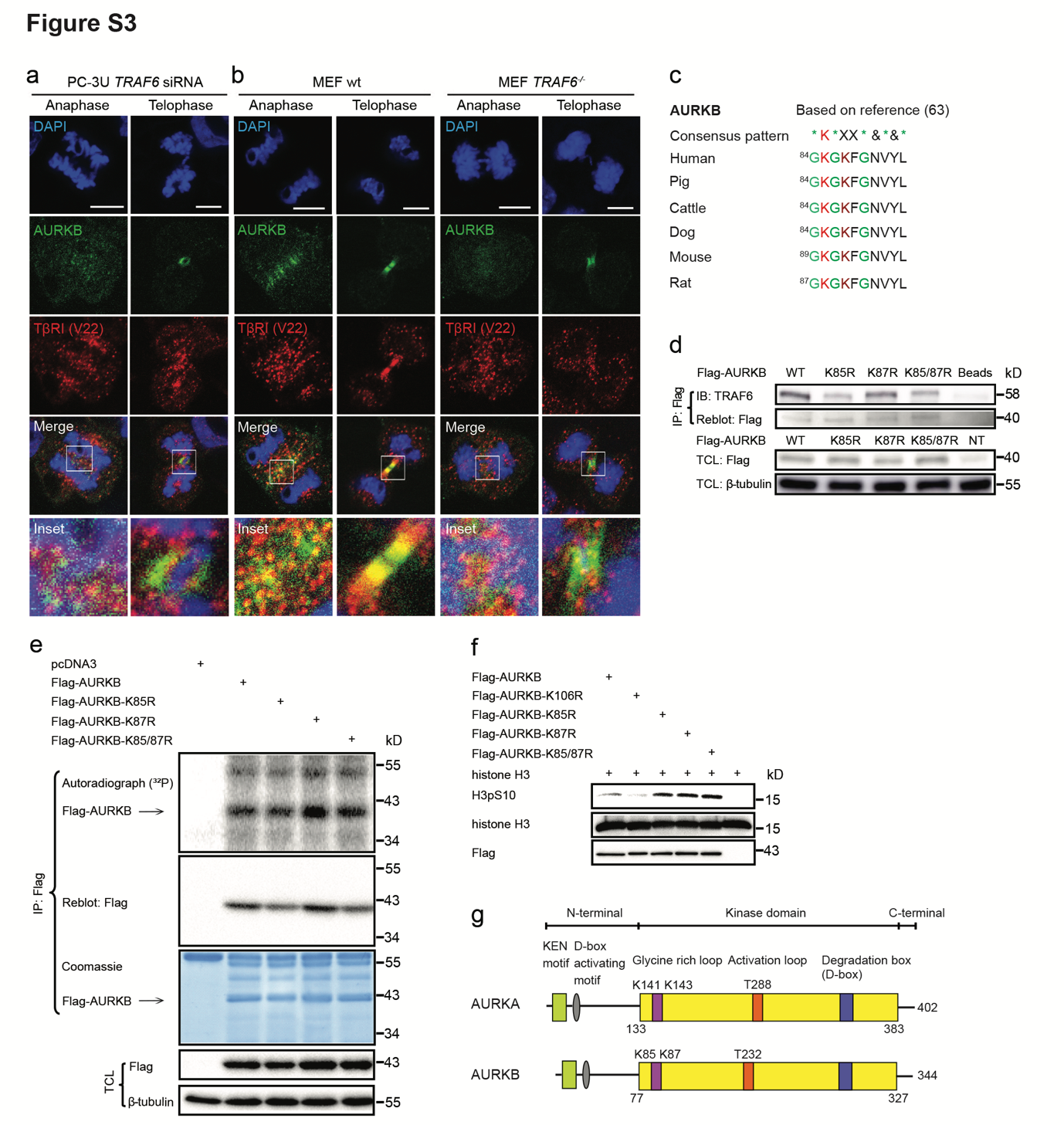


**Figure S3.** The colocalization between AURKB and TβRI is dependent on TRAF6 and characteristics of mutants AURKB. (a-b) Immunofluorescence showing reduced co-localization of AURKB and TβRI during mitosis when TRAF6 expression was decreased in PC-3U and MEF cells. (c) The consensus motif for ubiquitination by TRAF6 is present in AURKB in several species. Amino acids of the same type are labeled as (*) hydrophobic, (&) polar, (X) any amino acid residue. (K) is the acceptor lysine residue. (d) Lysates of PC-3U cells transfected with Flag-tagged WT and mutant AURKB were immunoprecipitated with a Flag antibody and then subjected to immunoblotting with a TRAF6 antibody, as indicated. (e-f) Immunoprecipitated Flag-AURKB or its mutants were subjected to the *in vitro* kinase assays. The expression of Flag-AURKB and its mutants and equal loading was controlled by immunoblotting aliquots of the Flag immunoprecipitates or total cell lysate (TCL), as indicated. Incorporated radioactivity was detected by a phosphorimager. Migration positions of phosphorylated proteins and total proteins detected after staining of gels with Coomassie Brilliant Blue are shown by arrows (e). Histone H3 was used as substrate and H3pS10 was detected by immunoblotting (f). (g) Structure of human sapiens AURKA and AURKB domains. AURKA and AURKB are constituted by an N-terminal domain which controls protein localization, a large and conserved kinase domain which contains the activation loop and degradation box (D-box) and a short C-terminal domain. AURKA and AURKB also contain a KEN motif and a D-box activating motif which regulates the degradation. Percentages of homology of the total amino acid sequence are evaluated at 57% between AURKA and AURKB. And percentages of homology of the kinase domain are evaluated at 71%. Note the K85 and K87 in the glycine rich loop in the beginning of AURKB kinase domain.


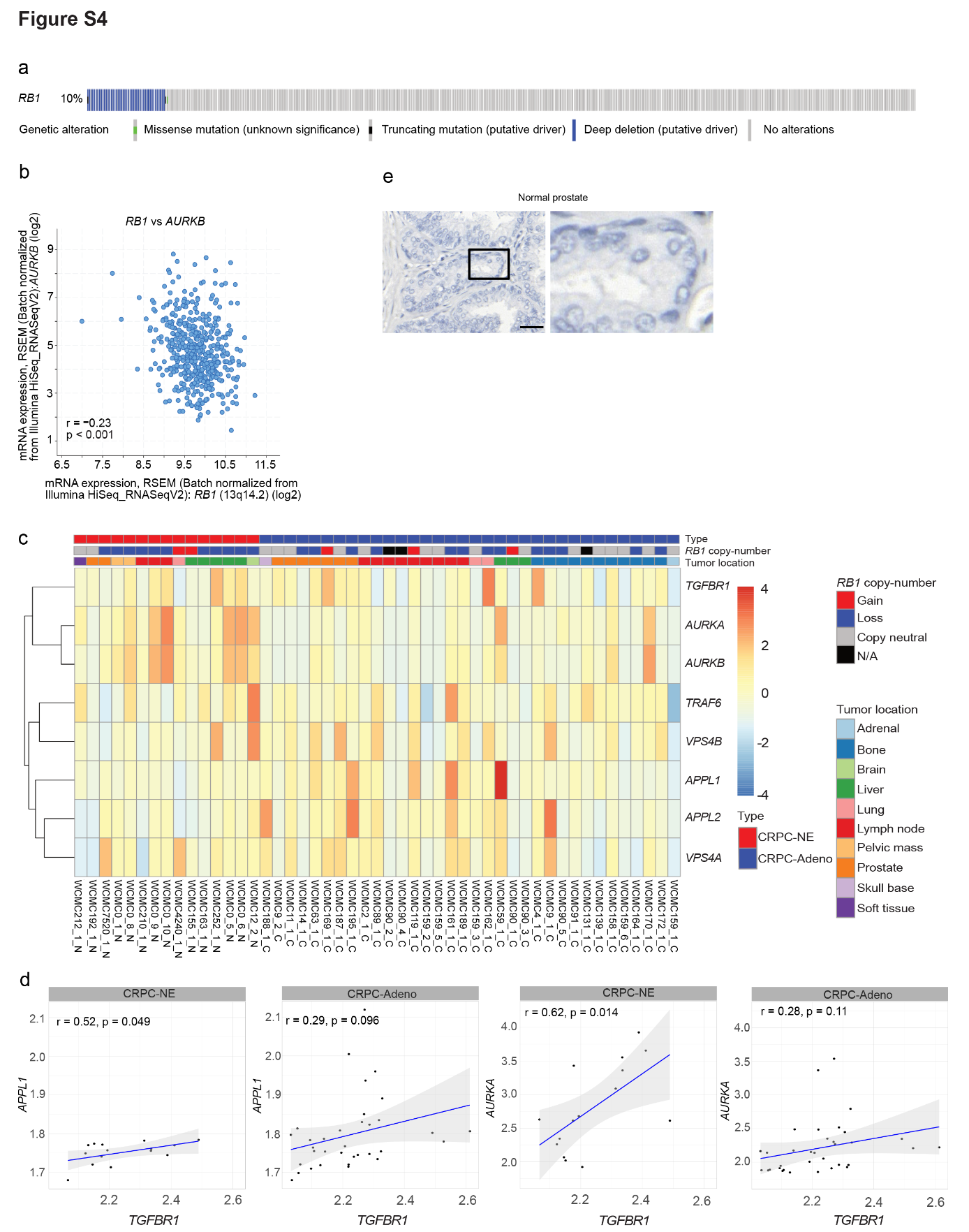


**Figure S4.** Relation between *RB1* and *AURKB* expression in prostate cancer and correlation between the expression of *APPL1*, *AURKA* and *TGFBR1* in CRPC. (a) *RB1* mutations in prostate cancer. (b) Negative correlation between expression of mRNA for *AURKB* and *RB1* in prostate cancer; Pearson correlation coefficient (r) is presented. Data were obtained from cBioPortal TCGA PanCancer Atlas databases. (c) Expression of eight genes of interest across 49 CRPC samples, with 15 CRPC-NE samples and 34 CPPC-Adeno samples, including both primary tumors and metastases. Samples are grouped first by their subtype and then by the tumor location. (d) Correlation of *APPL1, AURKA* and *TGFBR1* expression in CRPC-NE and CRPC-Adeno. Pearson correlation analysis are used for data analysis. (e) Lack of association between AURKB and TβRI in normal prostate tissue (brown dots), as determined by *in situ* PLA, serving as a negative control (no primary antibody was added). Scale bar, 50 µm.


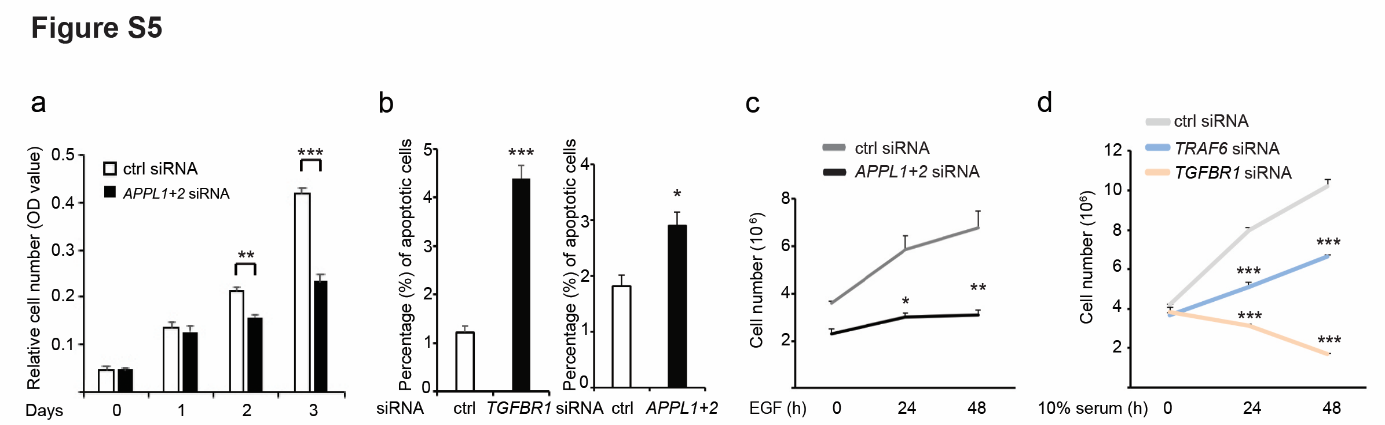


**Figure S5.** Effects of *APPL1/2*, *TGFBR1* and *TRAF6* on cell proliferation and survival. (a) PC-3U cells were treated with control (ctrl) siRNA or No.1 *APPL1/2* siRNA and subjected to MTT assay after different number of days in culture. (b) Apoptotic cells were counted among cells transfected with the different siRNAs. (c-d) Effects of silencing of the *APPL1/2* genes in PC-3U cells on EGF stimulated cell growth (c), and of silencing of the *TRAF6* or *TGFBR1* genes with siRNA on cell number stimulated by 10% FBS (d). N=3, Quantification shows mean±SEM [Student’s t-test, * P<0.05, ** P<0.01, ***P<0.001].
