# Supplementary material for: The ubiquitin-ligase TRAF6 and TGFβ type I receptor form a complex with Aurora kinase B contributing to mitotic progression and cytokinesis in cancer cells
Source: eBioMedicine. 2022 Jul 16;82:104155. doi: 10.1016/j.ebiom.2022.104155 (PMC9386726; doi:10.1016/j.ebiom.2022.104155)

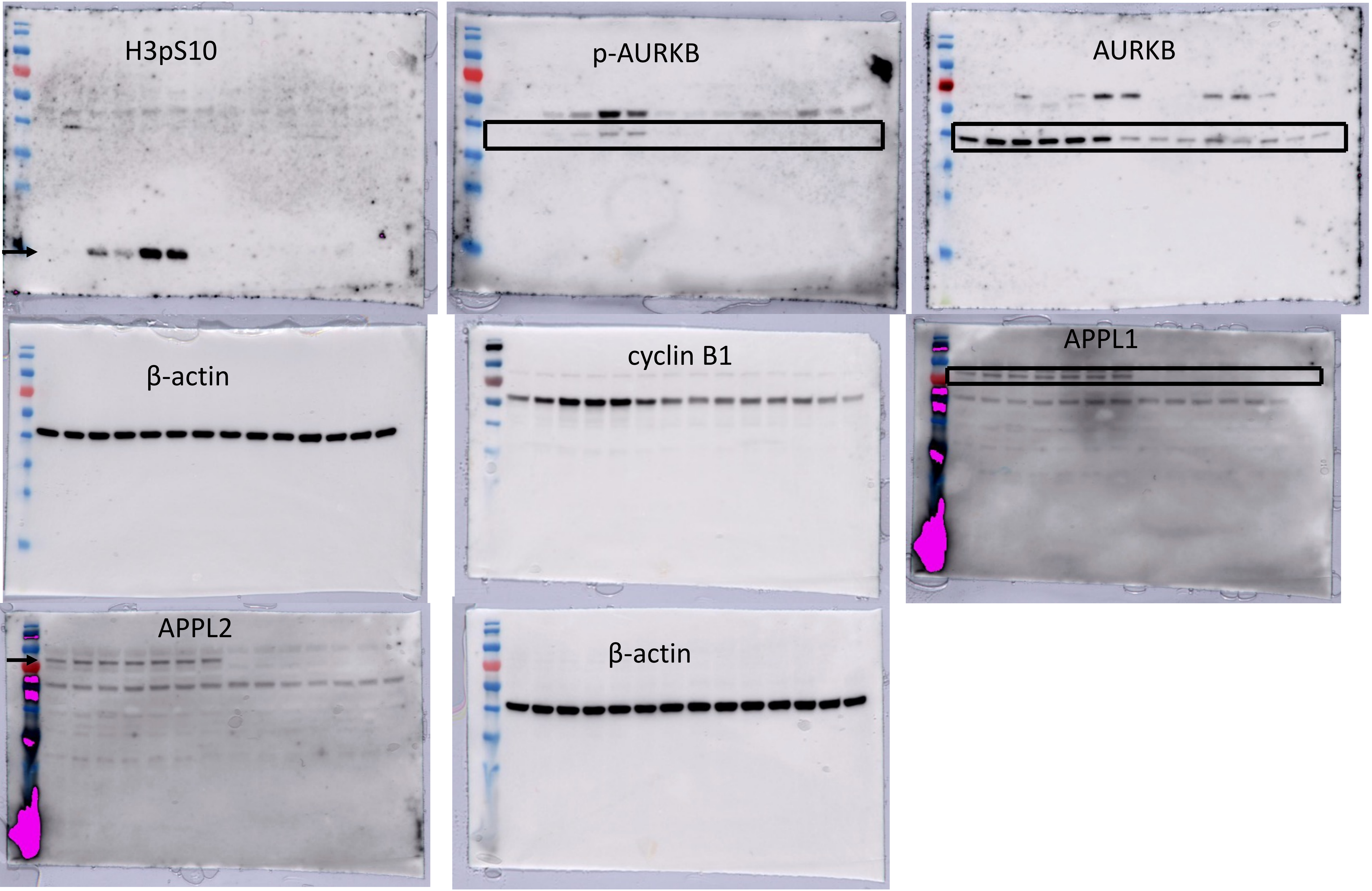

Figure 1c

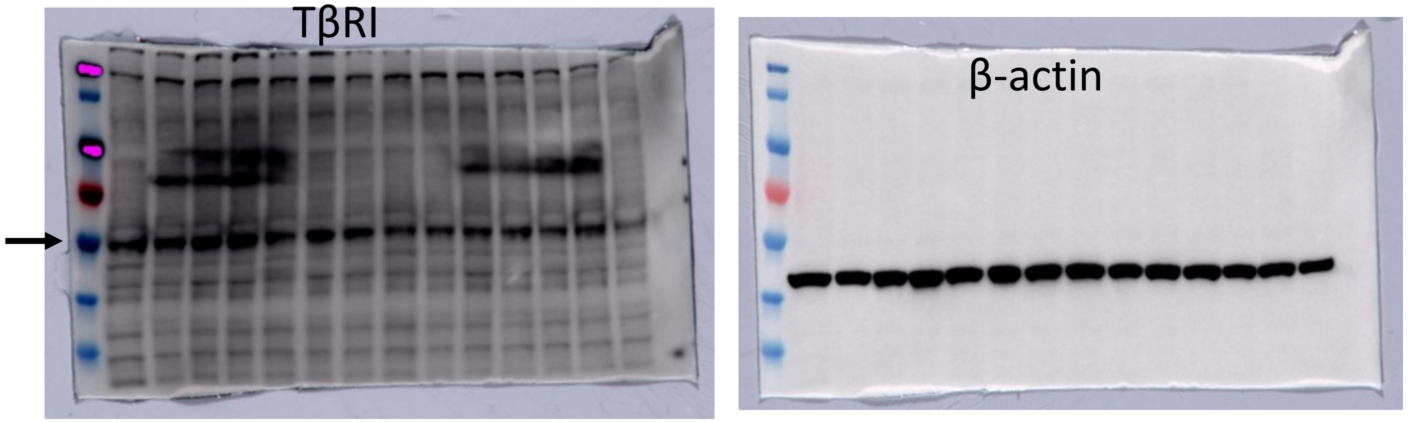

Figure 1c

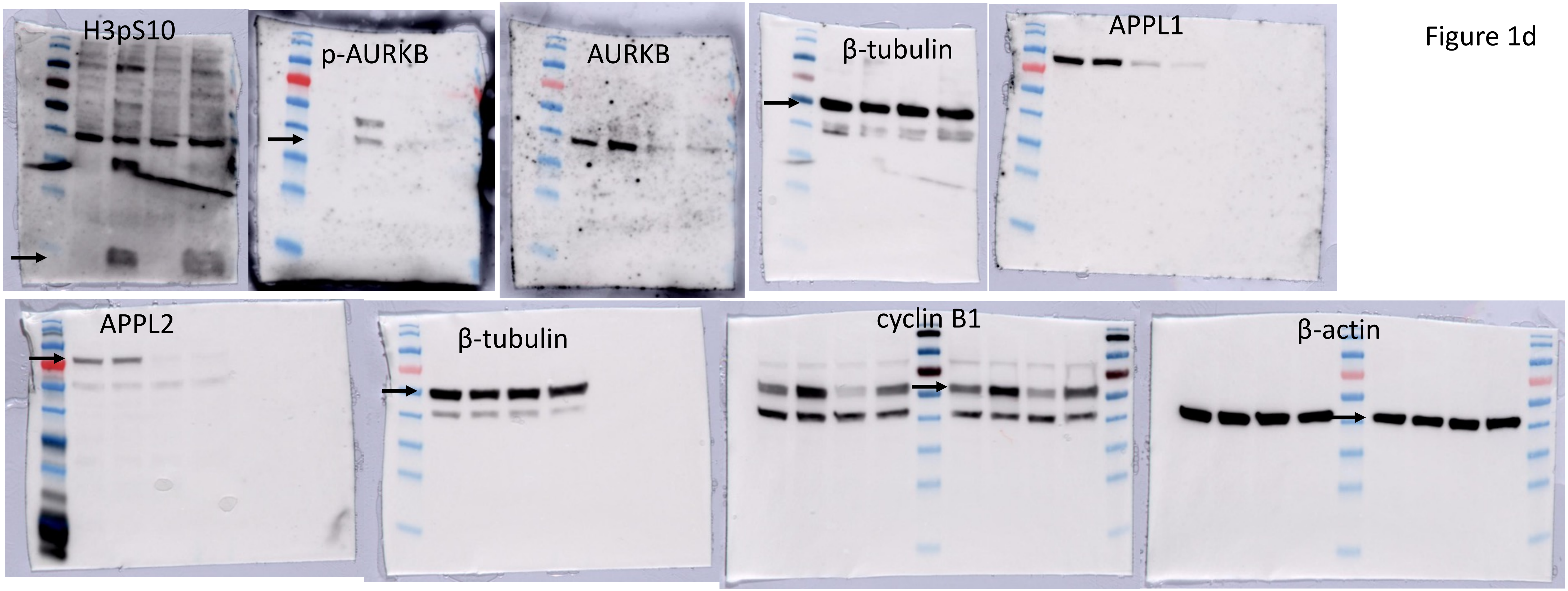

H3pS10
p-AURKB
AURKB
β-tubulin
APPL1
APPL2
β-tubulin
cyclin B1
β-actin

Figure 1d

Figure 1m

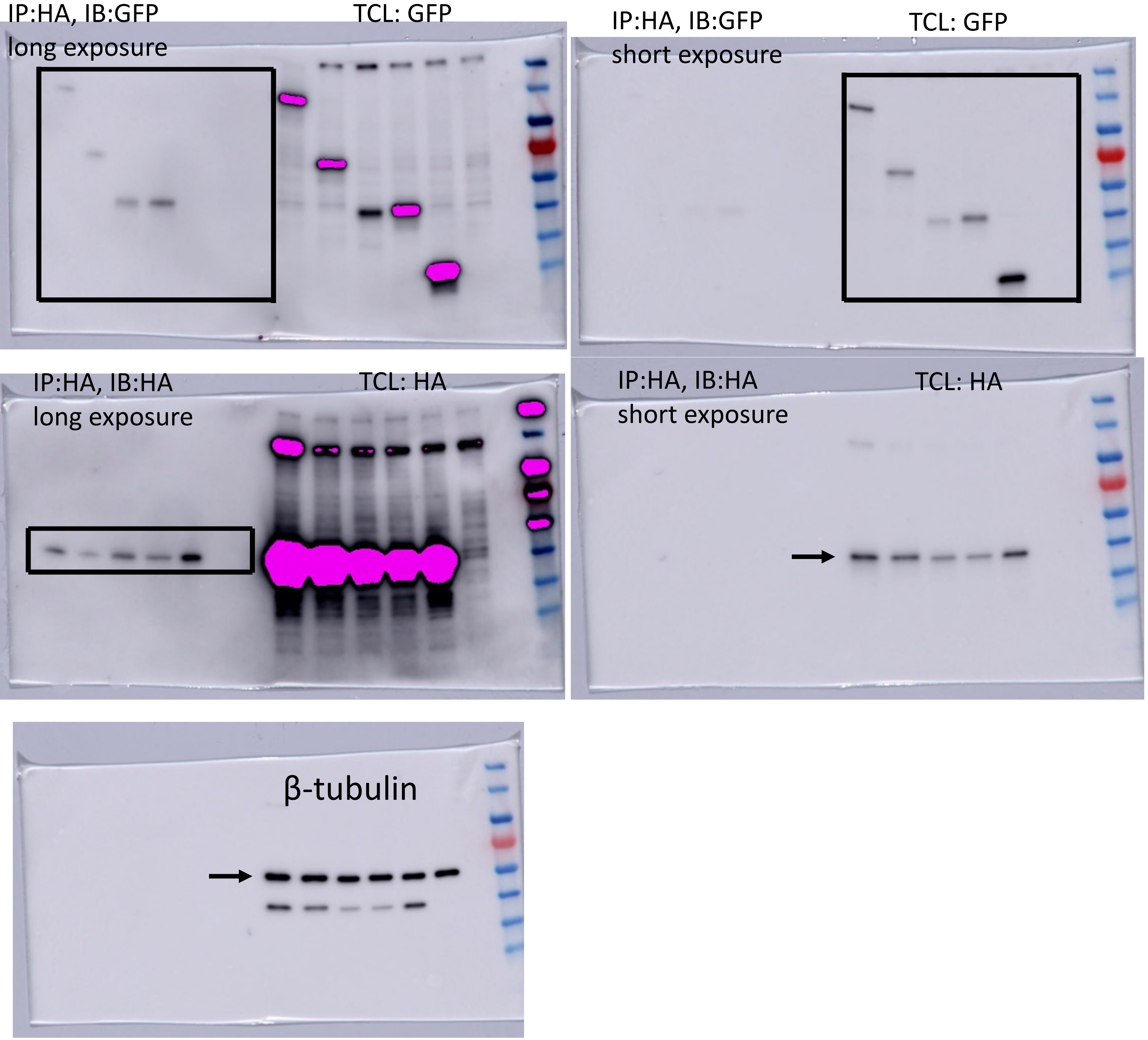
IP:HA, IB:GFP
long exposure
TCL: GFP
IP:HA, IB:GFP
short exposure
TCL: GFP
IP:HA, IB:HA
long exposure
TCL: HA
IP:HA, IB:HA
short exposure
TCL: HA
β-tubulin

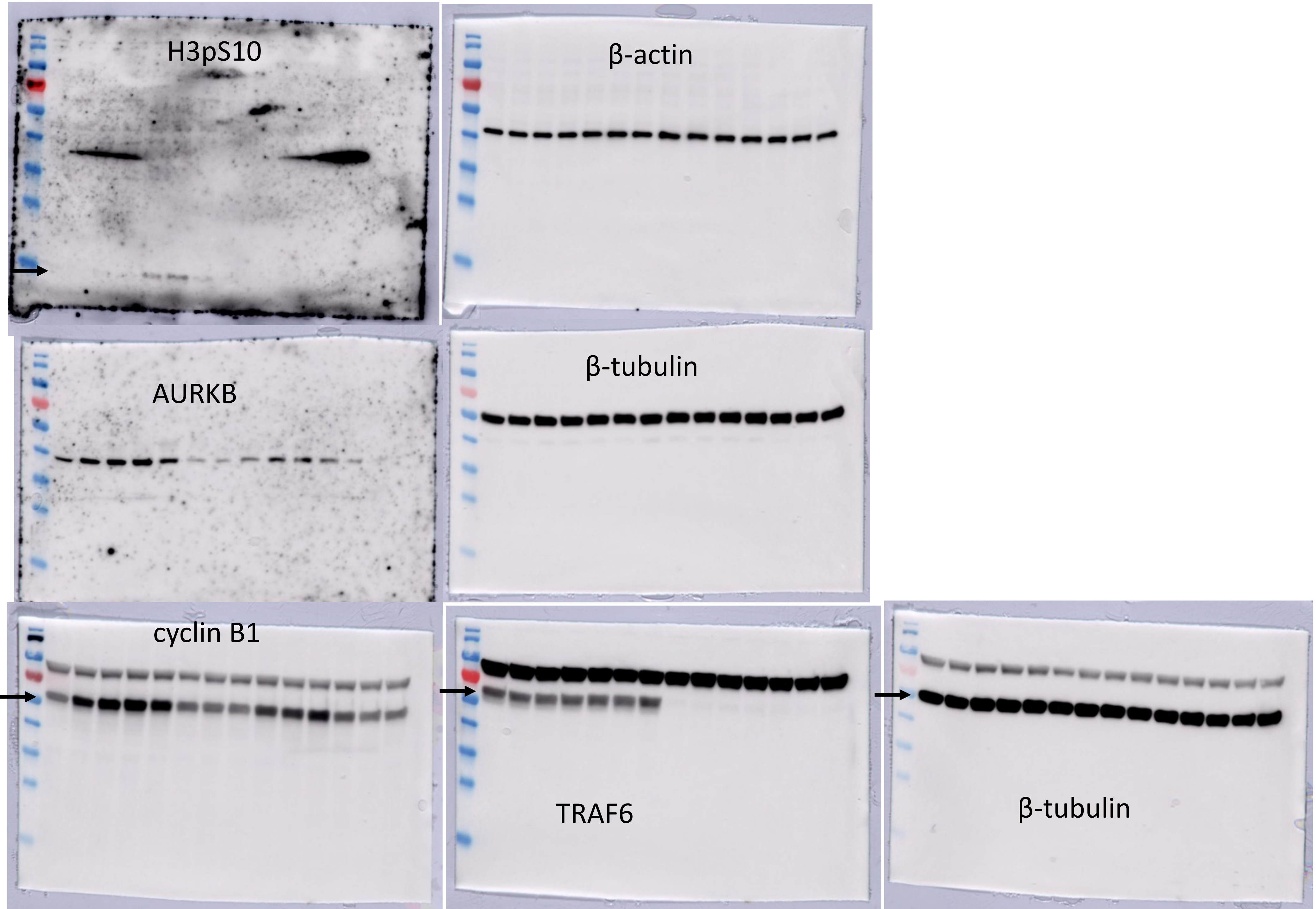

Figure 3a

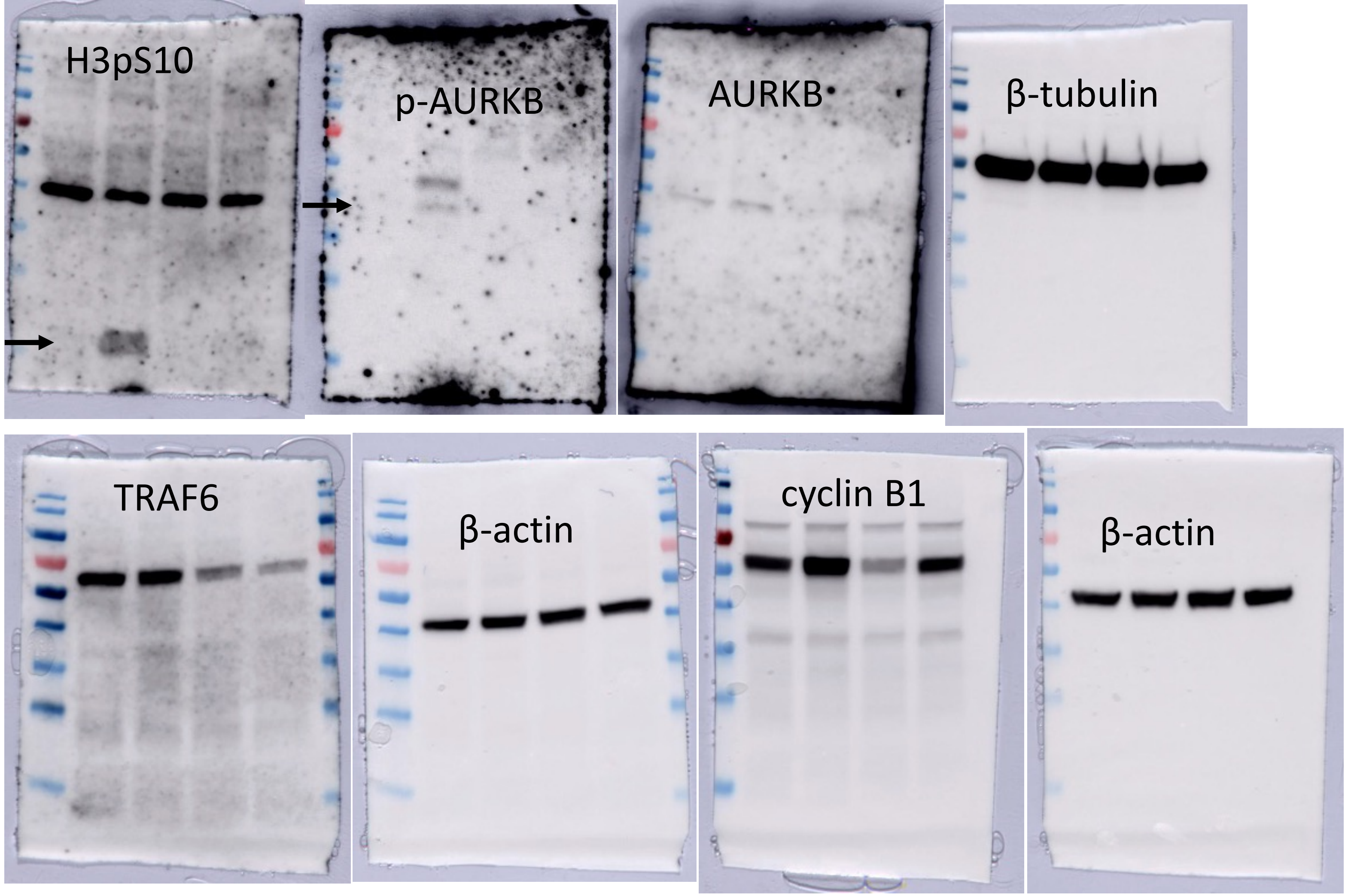
H3pS10
p-AURKB
AURKB
β-tubulin
TRAF6
β-actin
cyclin B1
β-actin

Figure 3b

Figure 3c

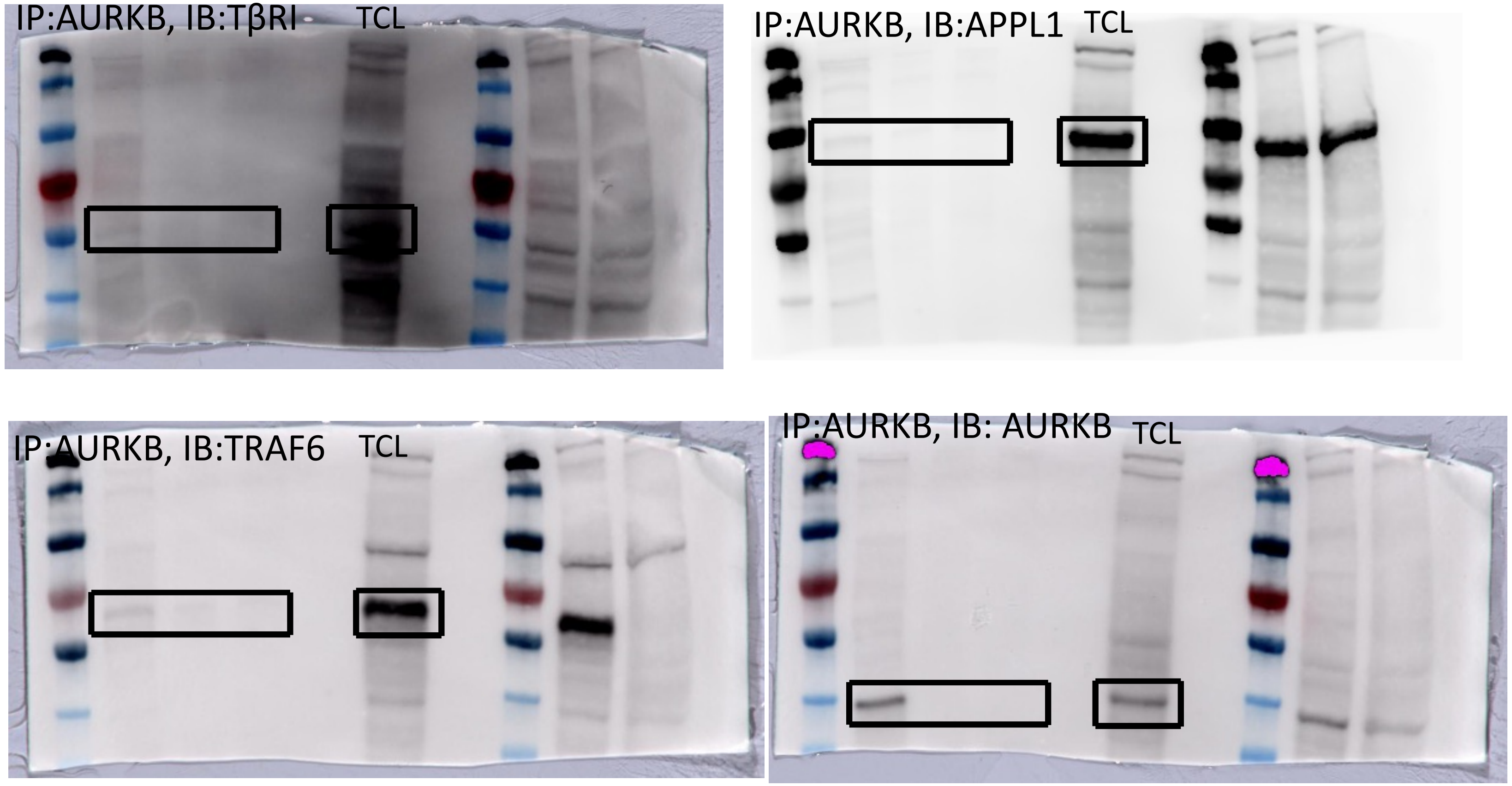

IP:AURKB, IB:TβRI
TCL
IP:AURKB, IB:APPL1
TCL
IP:AURKB, IB:TRAF6
TCL
IP:AURKB, IB: AURKB
TCL

Figure 3d

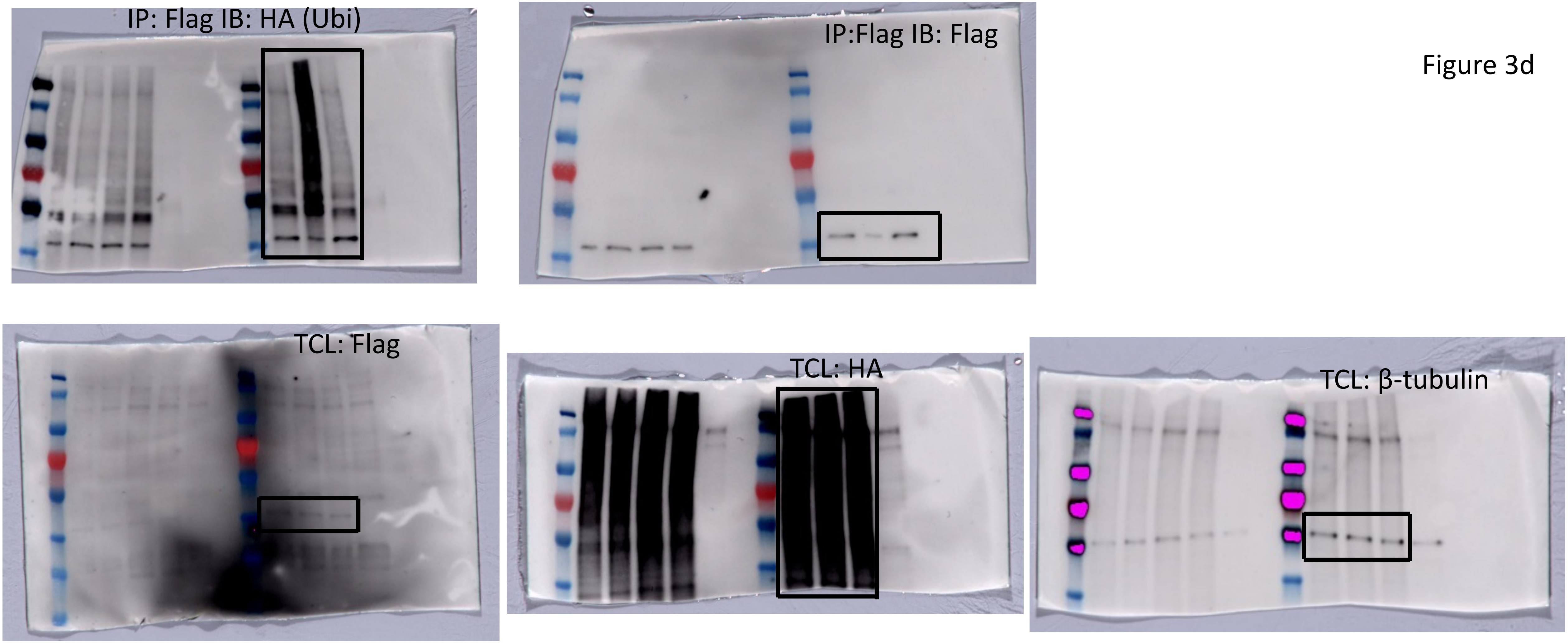

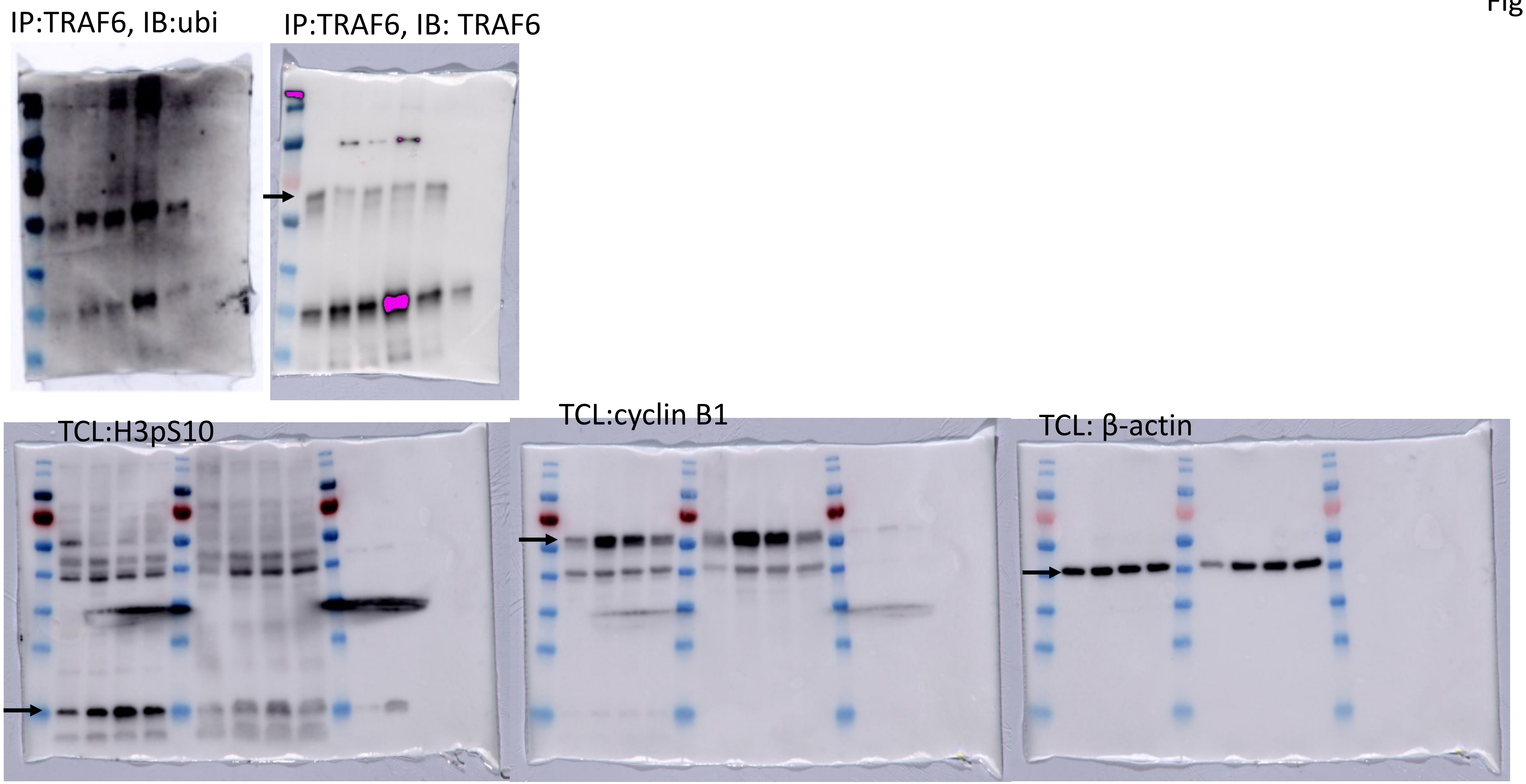
IP:TRAF6, IB:ubi
IP:TRAF6, IB: TRAF6
TCL:H3pS10
TCL:cyclin B1
TCL: β-actin

Figure 3e

Figure 3f

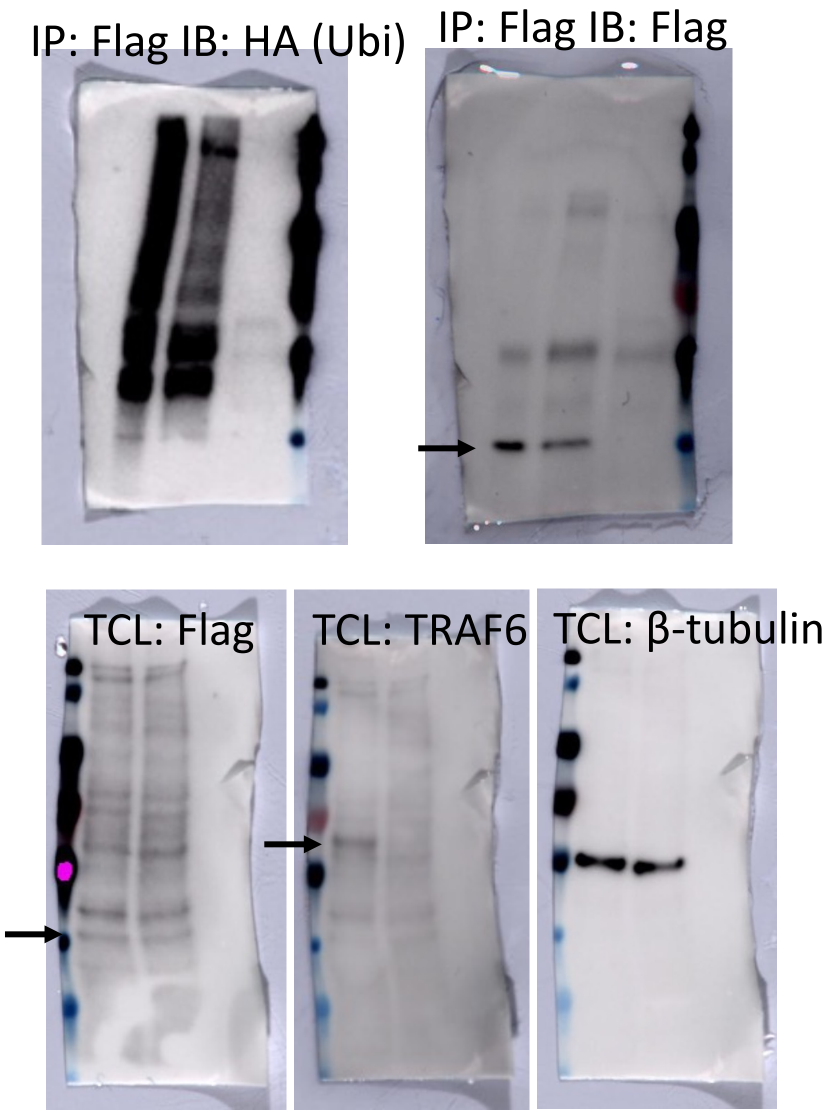
IP: Flag IB: HA (Ubi)
IP: Flag IB: Flag
TCL: Flag
TCL: TRAF6
TCL: β-tubulin

Figure 3g

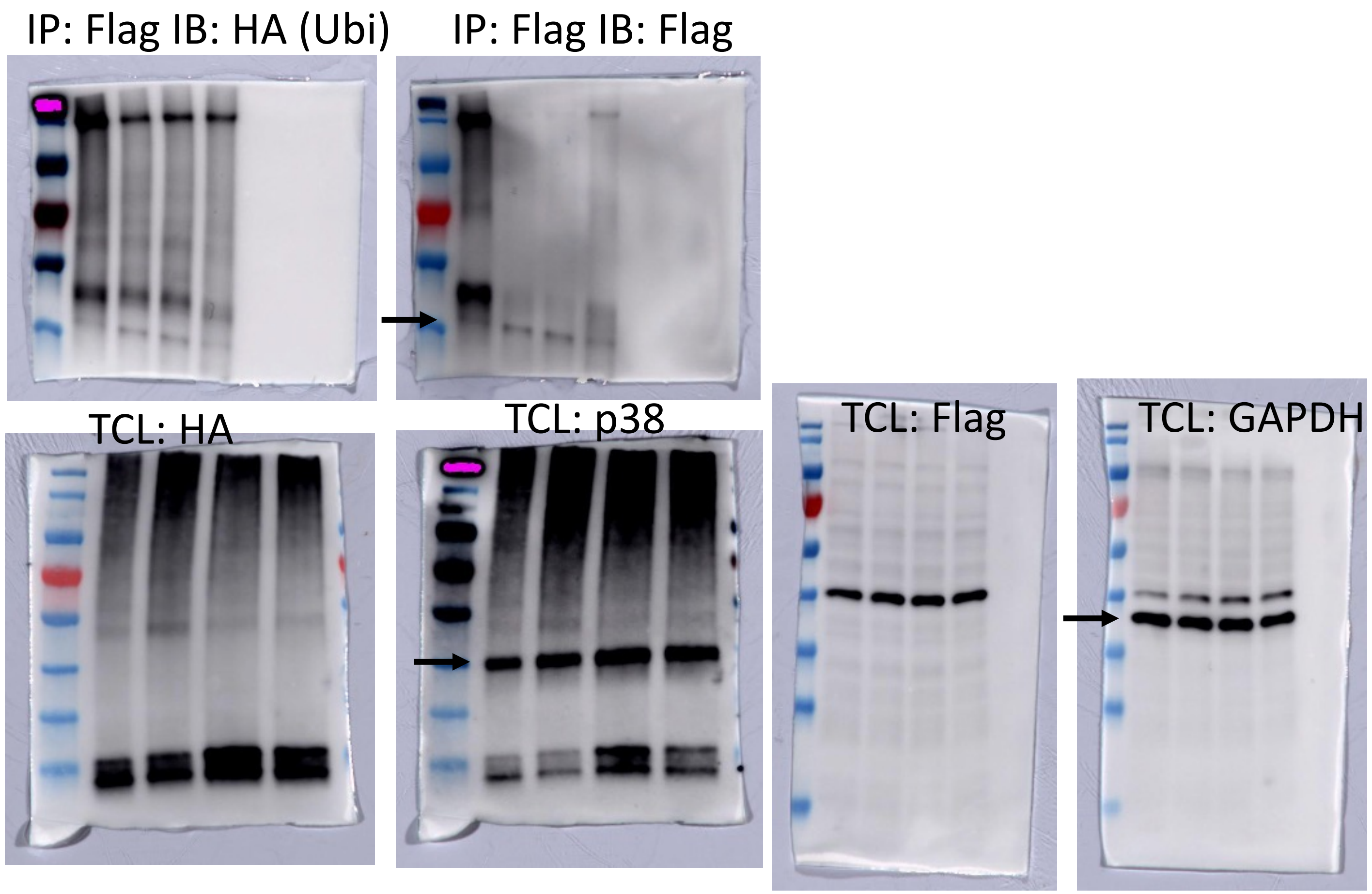
IP: Flag IB: HA (Ubi)
IP: Flag IB: Flag
TCL: HA
TCL: p38
TCL: Flag
TCL: GAPDH

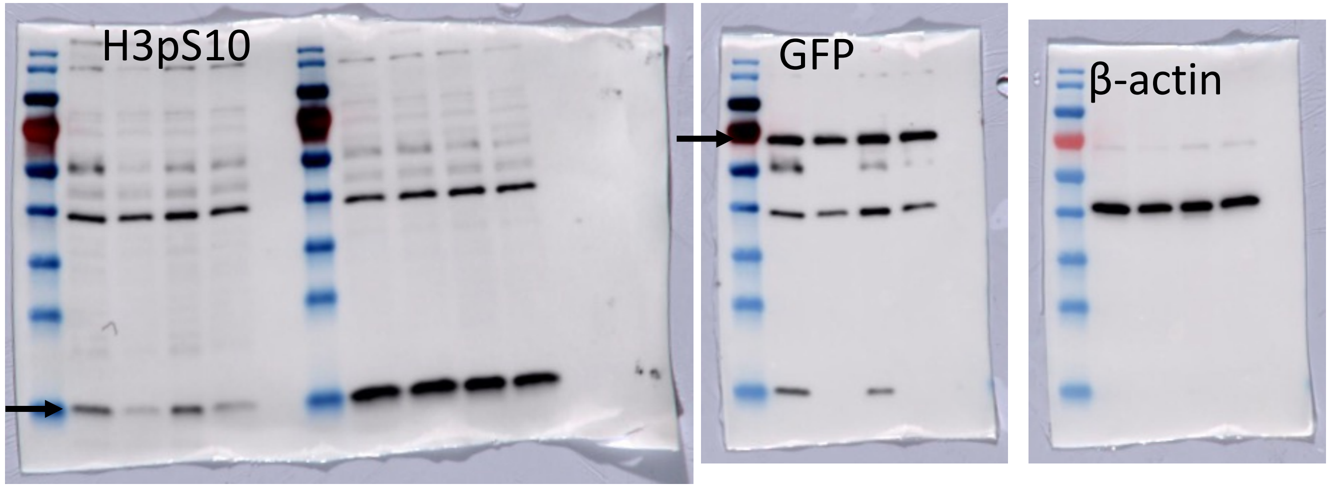

Figure 3h

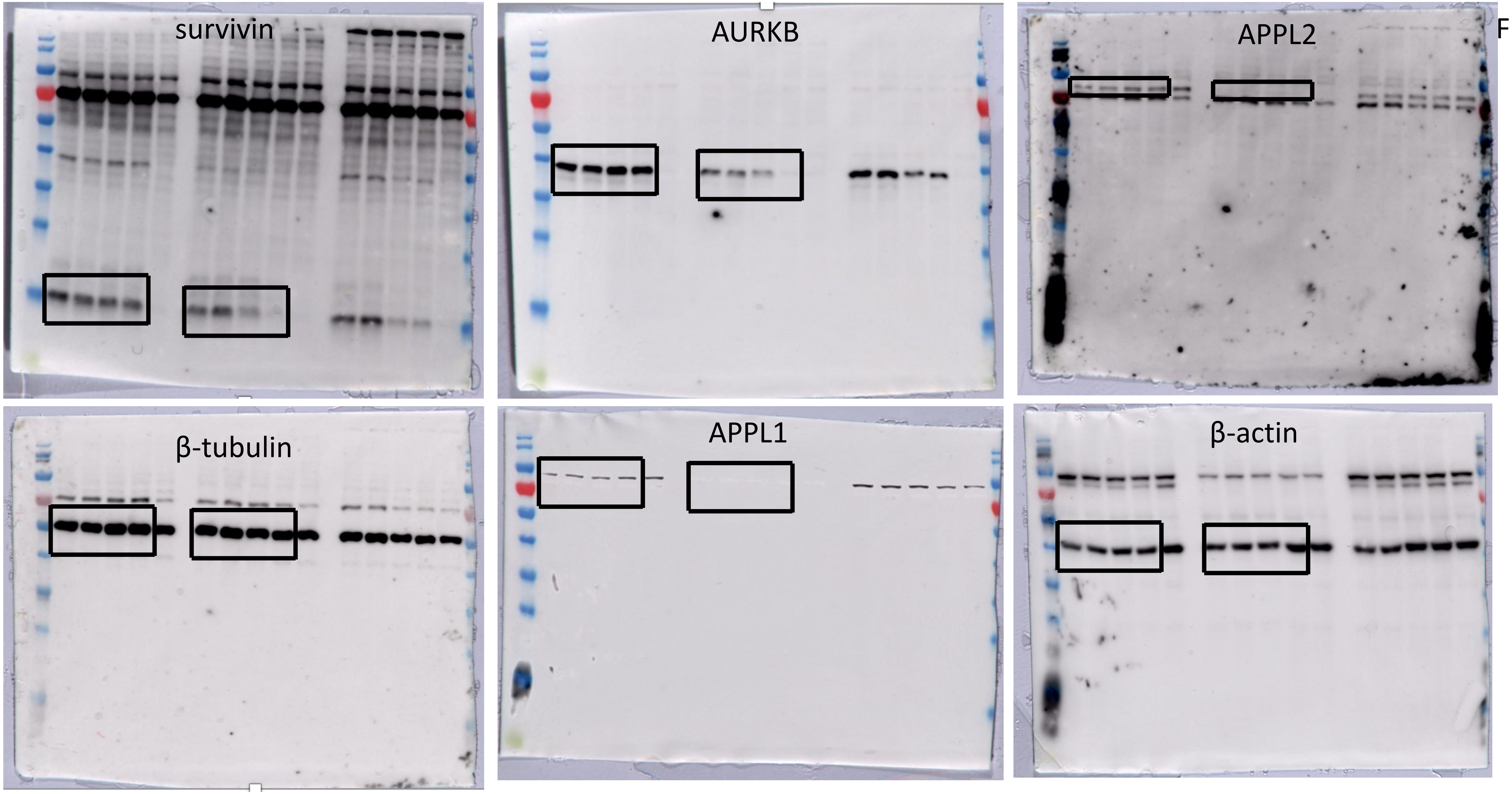

Figure S1c

Figure S3d

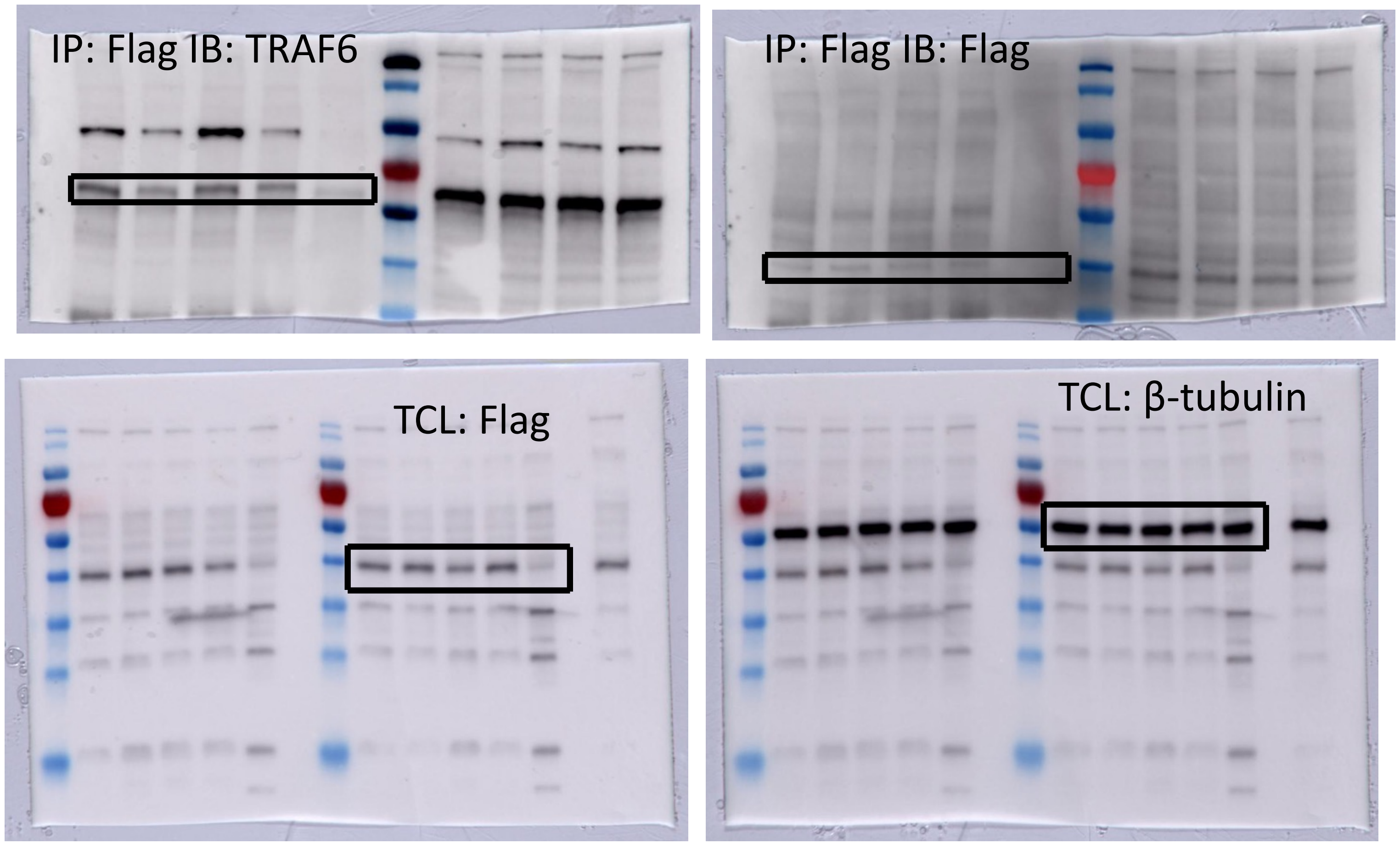

Figure S3e

Autoradiograph (^{32}P)

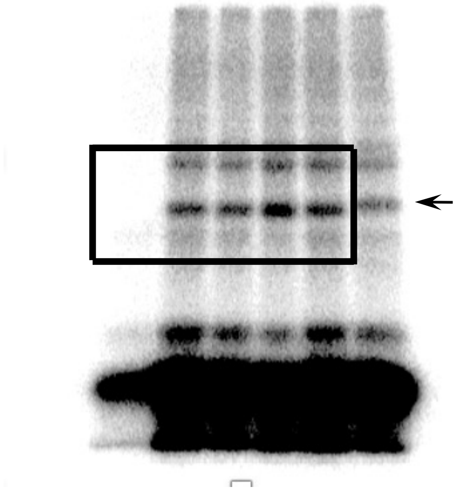

← Flag-AurKB

Commassie

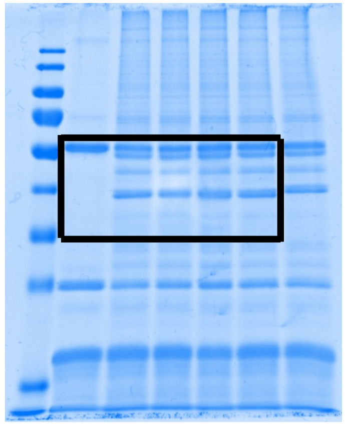

← Flag-AurKB

TCL: β-tubulin

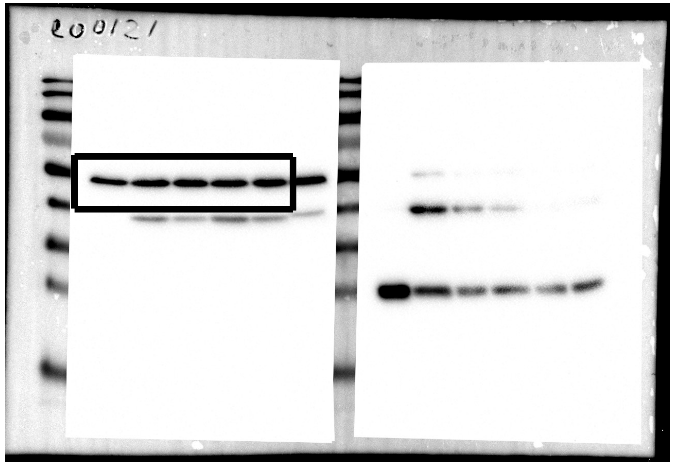

IP: Flag, IB: Flag   TCL: Flag

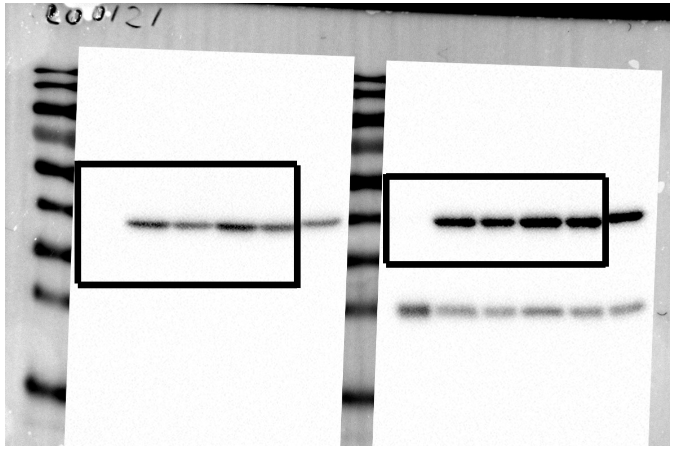

Figure S3f

H3pS10

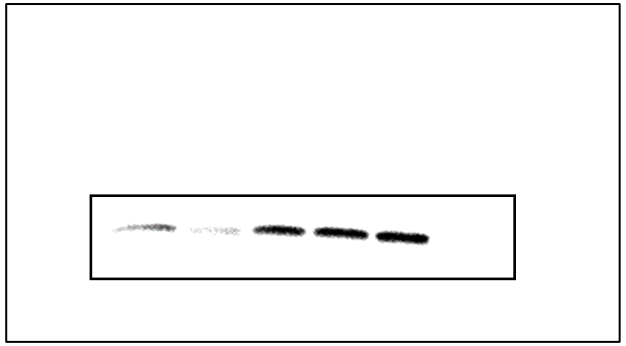

histone H3

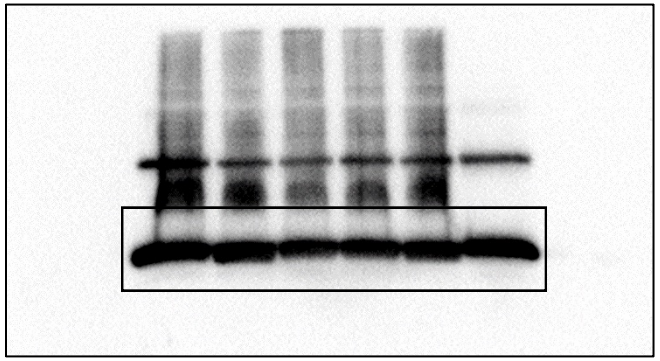

Flag

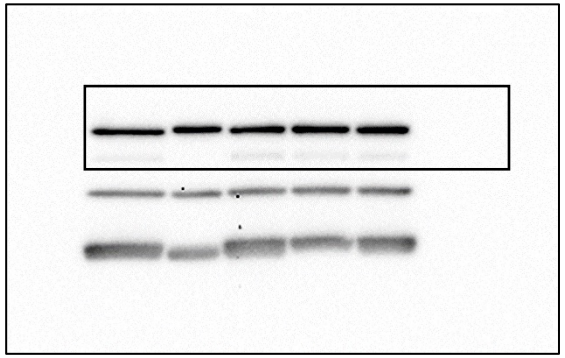

Supplement: Supplementary file 2 [file mmc2.pdf]
