# Supplementary figures and images for: The ubiquitin-ligase TRAF6 and TGFβ type I receptor form a complex with Aurora kinase B contributing to mitotic progression and cytokinesis in cancer cells
Source: eBioMedicine. 2022 Jul 16;82:104155. doi: 10.1016/j.ebiom.2022.104155 (PMC9386726; doi:10.1016/j.ebiom.2022.104155)

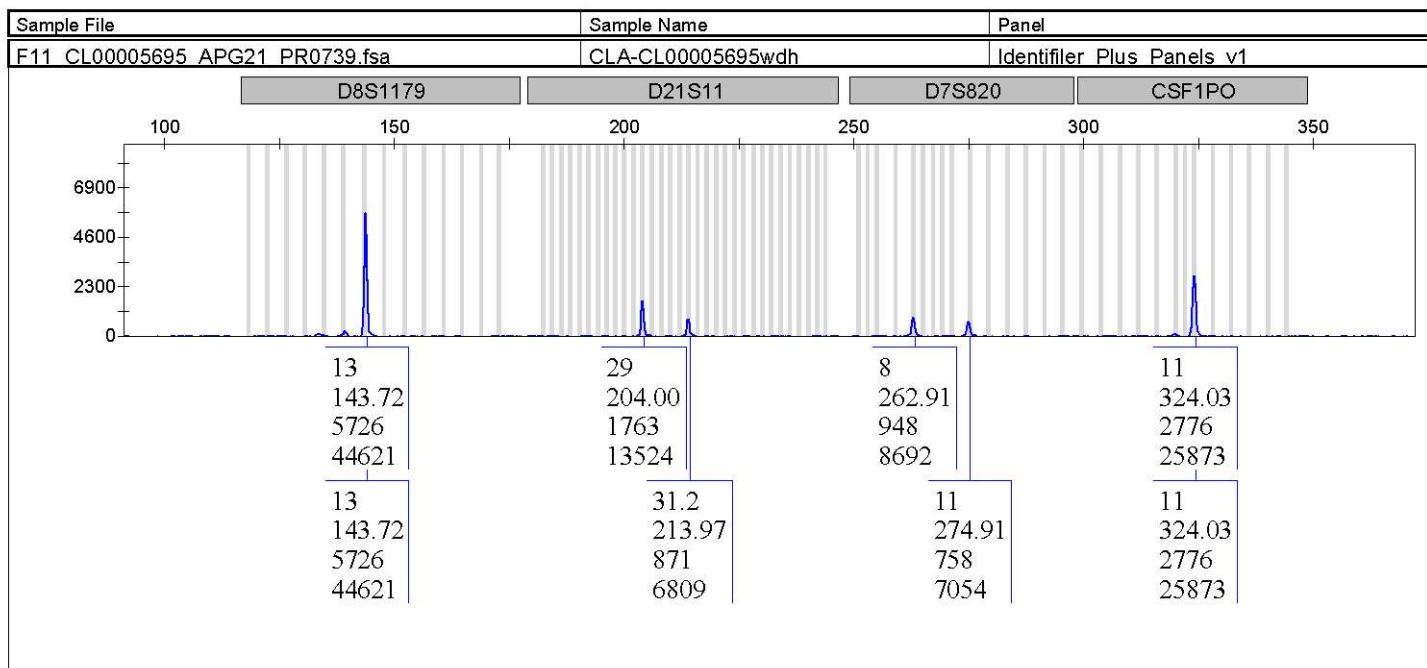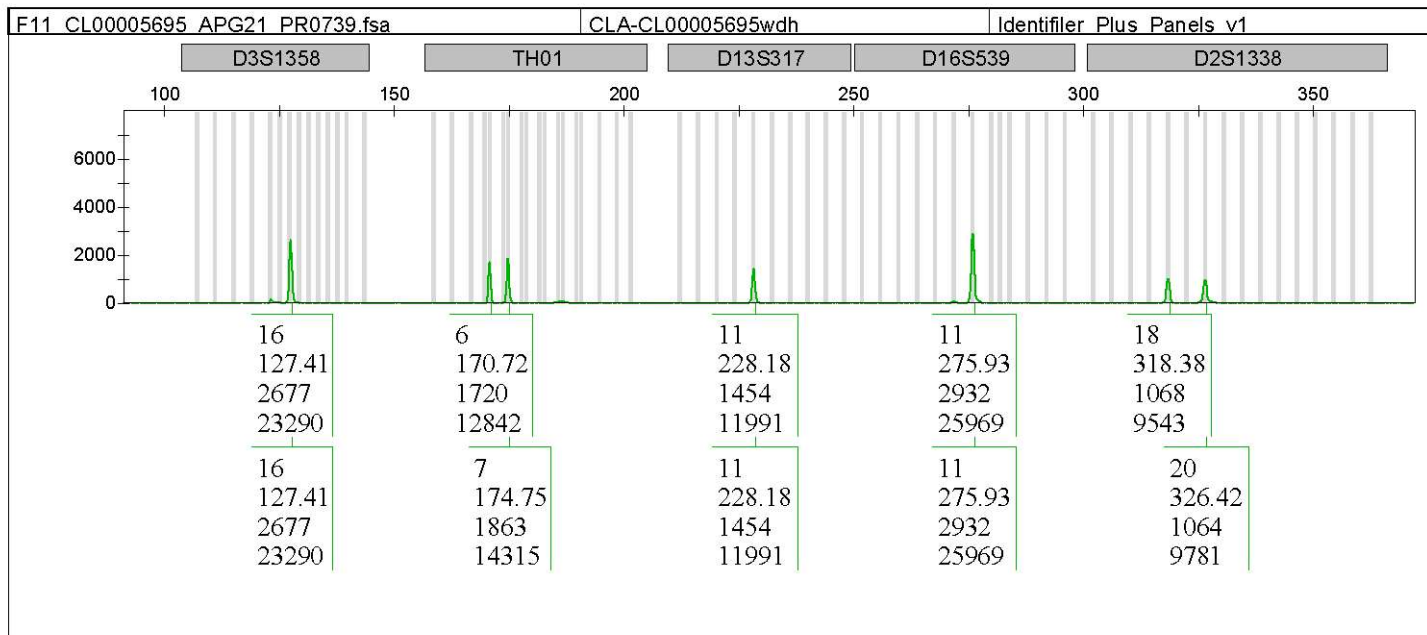

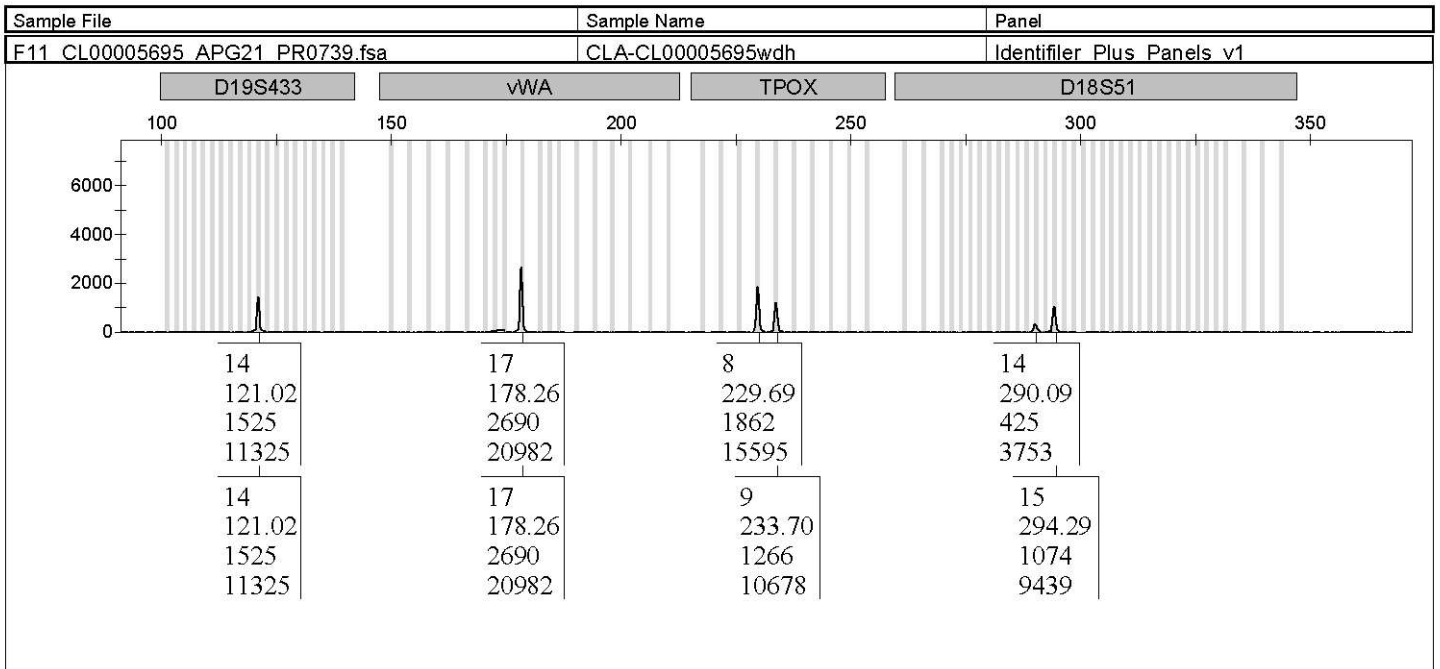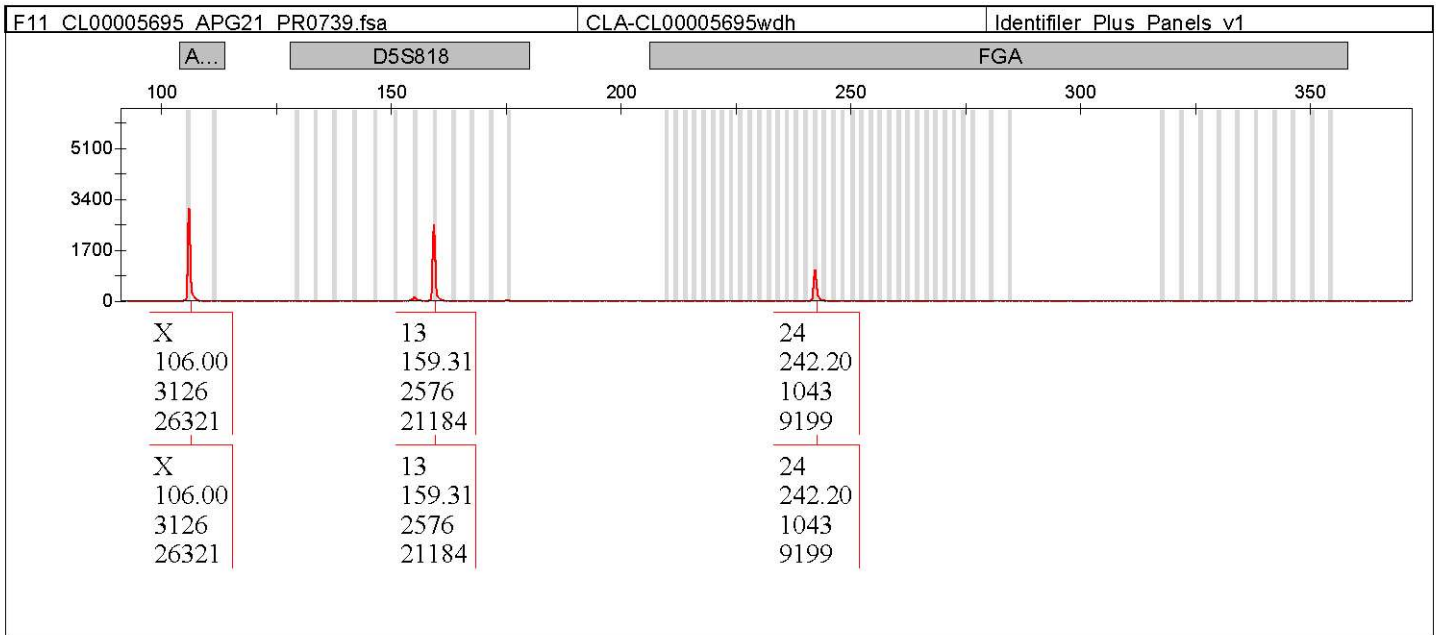

Supplement: Supplementary file 3 [file mmc3.pdf]
